# Formation of Large Native Sulfur Deposits Does Not Require Molecular Oxygen

**DOI:** 10.3389/fmicb.2019.00024

**Published:** 2019-01-25

**Authors:** Amanda L. Labrado, Benjamin Brunner, Stefano M. Bernasconi, Jörn Peckmann

**Affiliations:** ^1^Department of Geological Sciences, The University of Texas at El Paso, El Paso, TX, United States; ^2^Geological Institute, ETH Zürich, Zurich, Switzerland; ^3^Centrum für Erdsystemforschung und Nachhaltigkeit, Universität Hamburg, Hamburg, Germany

**Keywords:** native sulfur, sulfur reduction, sulfur formation, microbe, isotope, cryptic sulfur cycling, cryptic carbon cycling, methanogenesis

## Abstract

Large native (i.e., elemental) sulfur deposits can be part of caprock assemblages found on top of or in lateral position to salt diapirs and as stratabound mineralization in gypsum and anhydrite lithologies. Native sulfur is formed when hydrocarbons come in contact with sulfate minerals in presence of liquid water. The prevailing model for native sulfur formation in such settings is that sulfide produced by sulfate-reducing bacteria is oxidized to zero-valent sulfur in presence of molecular oxygen (O_2_). Although possible, such a scenario is problematic because: (1) exposure to oxygen would drastically decrease growth of microbial sulfate-reducing organisms, thereby slowing down sulfide production; (2) on geologic timescales, excess supply with oxygen would convert sulfide into sulfate rather than native sulfur; and (3) to produce large native sulfur deposits, enormous amounts of oxygenated water would need to be brought in close proximity to environments in which ample hydrocarbon supply sustains sulfate reduction. However, sulfur stable isotope data from native sulfur deposits emplaced at a stage after the formation of the host rocks indicate that the sulfur was formed in a setting with little solute exchange with the ambient environment and little supply of dissolved oxygen. We deduce that there must be a process for the formation of native sulfur in absence of an external oxidant for sulfide. We hypothesize that in systems with little solute exchange, sulfate-reducing organisms, possibly in cooperation with other anaerobic microbial partners, drive the formation of native sulfur deposits. In order to cope with sulfide stress, microbes may shift from harmful sulfide production to non-hazardous native sulfur production. We propose four possible mechanisms as a means to form native sulfur: (1) a modified sulfate reduction process that produces sulfur compounds with an intermediate oxidation state, (2) coupling of sulfide oxidation to methanogenesis that utilizes methylated compounds, acetate or carbon dioxide, (3) ammonium oxidation coupled to sulfate reduction, and (4) sulfur comproportionation of sulfate and sulfide. We show these reactions are thermodynamically favorable and especially useful in environments with multiple stressors, such as salt and dissolved sulfide, and provide evidence that microbial species functioning in such environments produce native sulfur. Integrating these insights, we argue that microbes may form large native sulfur deposits in absence of light and external oxidants such as O_2_, nitrate, and metal oxides. The existence of such a process would not only explain enigmatic occurrences of native sulfur in the geologic record, but also provide an explanation for cryptic sulfur and carbon cycling beneath the seabed.

## Introduction

### Epigenetic Native Sulfur Deposits

**FIGURE 1:**
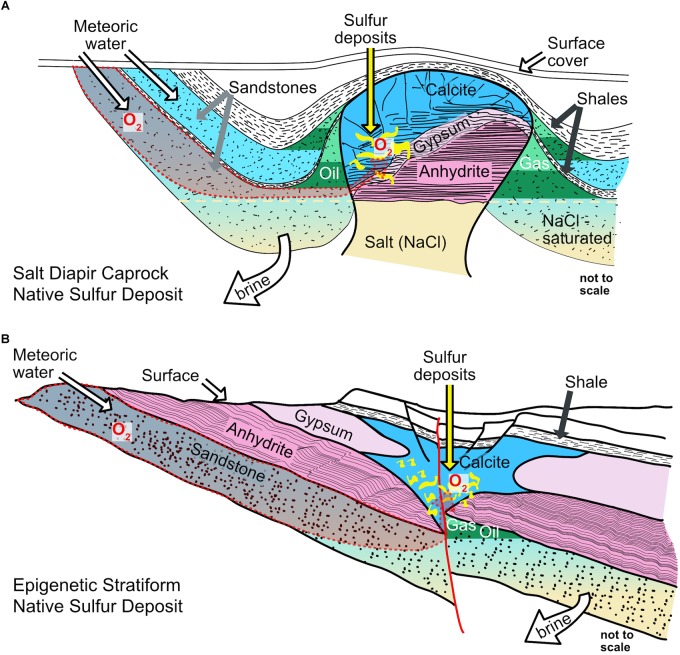
Sketches of classical epigenetic salt diapir caprock and stratabound sulfur deposit types. **(A)** Oxygen is delivered to the site where native sulfur is formed by infiltration of meteoric water. On its journey to the location where native sulfur is formed, O_2_ has to pass hydrocarbon bearing strata. Solubility of O_2_ may decrease with depth due to increased salinity. To maintain inflow of meteoric waters, the brine must be removed (modified from Ruckmick et al., 1979). **(B)** Oxygen is delivered to the site where native sulfur is formed by infiltration of meteoric water. On its journey to the location where native sulfur is formed, O_2_ takes the same route as the hydrocarbons. Solubility of O_2_ may decrease with depth due to increased salinity. To maintain inflow of meteoric waters, the brine must be removed (modified from Ruckmick et al., 1979).

Native sulfur is formed by a number of abiotic and biological processes in a multitude of settings such as deeply buried sediments by thermochemical sulfate reduction (Warren, 2006), seafloor hydrothermal systems (Butterfield et al., 2011; Seewald et al., 2015), at volcanoes (e.g., in Chile; Ferraris and Vila, 1990), at arctic glaciers (Grasby et al., 2003), in lake sediments (Philip et al., 1994; Lindtke et al., 2011), in shallow marine sediments (e.g., through the giant sulfur bacterium *Thiomargarita namibiensis*; Schulz and Schulz, 2005), in sulfidic cave systems (e.g., incomplete sulfide oxidation by *Sulfurovum-like Epsilonproteobacteria*; Hamilton et al., 2015), or at the seafloor as filamentous sulfur (i.e., by *Beggiatoa*; Jørgensen et al., 2010 or by *Arcobacter*; Wirsen et al., 2002; Sievert et al., 2007). On a geologic timescale, many of the native sulfur accumulations in these environments are transient. Upon burial native sulfur can be reduced to sulfide, whereas extended periods of exposure to oxic conditions lead to its oxidation to sulfate. Indeed, evidence for large filamentous sulfide-oxidizing bacteria such as *Beggiatoa* or *Thioploca* is available for only few examples that represent syngenetic native sulfur formation (Druckman et al., 1994; Burhan et al., 2002). Syngenetic native sulfur deposits are formed at the same time as the strata that host them, whereas epigenetic native sulfur deposits (ENSDs) are emplaced after the formation of the host rocks. There are two categories of ENSDs: caprock and stratabound deposits (Ruckmick et al., 1979). When interpreted to have formed to a large extent by biological processes, they are referred to as bioepigenetic. The native sulfur in bioepigenetic deposits typically replaces a sulfate-bearing host rock, such as gypsum (CaSO_4_ ⋅ 2H_2_O) or anhydrite (CaSO_4_), and is commonly associated with authigenic carbonate minerals, such as calcite, aragonite, or dolomite. In stratabound deposits, native sulfur replaces sulfate-rich strata that were emplaced during the deposition of evaporite rocks, whereas in caprock deposits, native sulfur replaces sulfates that had accumulated at the crest of a salt diapir through preferential dissolution of sodium chloride salts (Figure 1; Davis and Kirkland, 1979; Ruckmick et al., 1979; Klimchouk, 1997). Ultimately, the driver for the replacement of sulfate-bearing host rocks is the availability of hydrocarbons and other organic compounds (e.g., carboxylic acids), which can be supplied by the migration of oil and natural gas. The organic compounds fuel sulfate reduction by coupling it to carbon oxidation, a process that yields carbonate minerals and reduced sulfur species.

Bioepigenetic native sulfur deposits can be enormous, with sizes reaching 89 and 500 million tons of native sulfur for caprock and stratabound deposits, respectively (Long, 1992a,b). It has been estimated that approximately four barrels of oil (∼560 kg) or 72,000 cubic feet of methane (∼1300 kg) are needed to form one metric ton of native sulfur (Ruckmick et al., 1979). At Damon Mound, Texas, which with 0.14 million tons of native sulfur (Long, 1992a) is a comparably small caprock deposit, this would correspond to 0.56 million barrels of crude oil. However, based on a minimum weight estimate of carbonate caprock of 32.7 million metric tons, and assuming that the carbon in the rock was derived from oil, the consumption was approximated to be 34.2 million barrels of crude oil (approximately 4.9 million metric tons; Sassen et al., 1994). This demonstrates that calculations of hydrocarbon consumption based on the presence of native sulfur are minimum estimates. While such numbers may appear staggering, a comparison shows that oil and gas reservoirs can supply the required hydrocarbon volumes. Damon Mound, which compared to other salt domes in the United States Gulf Coast salt province is considered a small oil reservoir (Sassen et al., 1994), yielded a cumulative oil production of 21.6 million barrels (Halbouty, 1979), demonstrating that enough hydrocarbons can by supplied to these environments.

**FIGURE 2:**
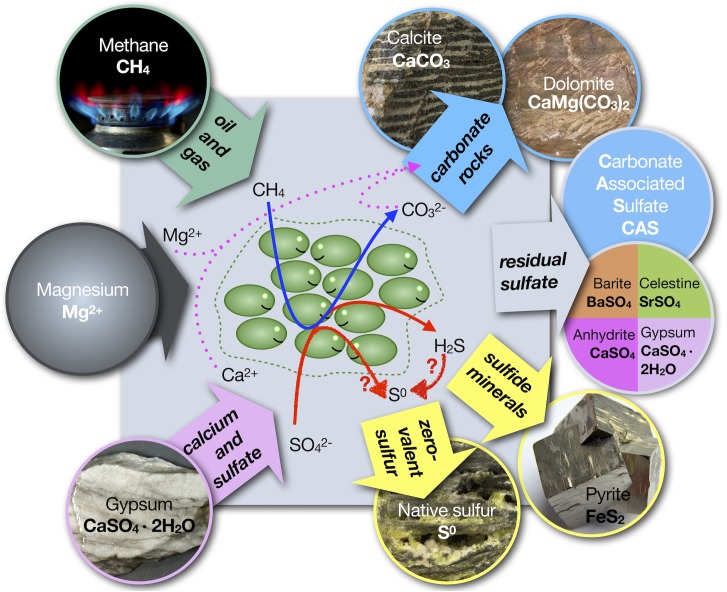
Schematic of genesis of sulfide, native sulfur and carbonate minerals from hydrocarbons and calcium sulfate minerals. The red arrows with question marks indicate that native sulfur is either formed by an unknown microbial pathway or that an unknown oxidant is needed for the conversion of sulfide into native (zero-valent) sulfur. In cases where dolomite is formed, there must be a (unknown) source of magnesium. Dissolved sulfate in system (gray box) can become trapped in newly formed carbonate minerals as carbonate associated sulfate (CAS).

Arguably, from a biogeochemical perspective, ENSDs are the most intriguing native sulfur deposits. Since the recognition that anaerobic bacteria may have generated the native sulfur deposits in Sicily by Hunt (1915), a problem vexes scientists to this day: presumably, sulfide is the only product of dissimilatory sulfate reduction and a subsequent oxidative step is required to generate native sulfur (Figure 2). The seemingly easy way out of this problem, the circumstance that sulfide is readily oxidized to zero-valent sulfur in the presence of molecular oxygen (O_2_), comes with two caveats: (1) there is no geochemical proof for the involvement of O_2_ in the genesis of the ENSDs; and (2) for some, if not most, ENSDs it is difficult to conceive how O_2_ could be supplied in the required quantities without inhibiting dissimilatory sulfate reduction. For example, to generate the largest native sulfur caprock deposit with 89 million tons of native sulfur (Boling dome; Long, 1992a), approximately 44 million tons or 1.4 ⋅ 10^12^ moles of O_2_ would be required, not including O_2_ lost to hydrocarbon oxidation. If such a supply is provided by oxygenated water to the subsurface and assuming a solubility of 250 μmol O_2_ per liter freshwater, this corresponds to approximately 5,100 km^3^ of oxygenated water, or for ease of comparison, to 9 months of draining of the Amazon River. Despite these concerns (e.g., Lindgren, 1913; Feely and Kulp, 1957; Parafiniuk, 1989; Kirkland, 2014), it has become widely accepted that oxidation of sulfide with O_2_ is required for the formation of bioepigenetic native sulfur deposits (Ruckmick et al., 1979; Kyle and Posey, 1991; Machel, 1992; Klimchouk, 1997). This notion has been eloquently summarized by Machel (1992): “Economically viable deposits of native sulfur usually are formed by only one process: inorganic oxidation of H_2_S by molecular oxygen.” The disparity between the paradigm that O_2_ is required and the absence of direct evidence that O_2_ is available in the needed quantities to drive the process resulted in a conundrum that has now persisted for over 100 years.

### A Geological Problem in Search of a Microbiological Solution

In the last three decades, the fields of environmental microbiology and biogeochemistry have made great progress. The new insights on microbial sulfur transformations and improved understanding of light stable isotope systematics and fractionation prompt us to re-evaluate the paradigm that presence of O_2_ is a prerequisite for the formation of ENSDs. In Section “Challenges With O_2_ as the Electron Acceptor for Sulfide Oxidation,” we first review the issues that arise with O_2_ being a prerequisite for the formation of native sulfur deposits. In Section “Alternatives to the Oxidation of Sulfide With O_2_ for the Genesis of Epigenetic Native Sulfur Deposits,” we introduce potential microbiological solutions to this geological problem. These solutions include metabolisms related to methylated compounds, ammonium transformations, and sulfur comproportionation reactions, which may allow for native sulfur genesis and accumulation on a geologic scale in the absence of O_2_ or other oxidants, such as nitrate or metal oxides. We demonstrate that (1) these processes are thermodynamically feasible, (2) response to sulfide stress could be the trigger for organisms to shift from the most energy yielding process (sulfide generation) to a less energy yielding catabolism (native sulfur formation) because it is more sustainable, and (3) environmental and experimental data indicate that the hypothesized processes indeed exist.

## Challenges With O_2_ as the Electron Acceptor for Sulfide Oxidation

Before we introduce the microbiological solutions to the O_2_ conundrum of ENSD genesis, we critically evaluate six central concepts of the current models:

(1) Are there examples of ENSDs where there is direct evidence for the presence or absence of O_2_?
(2) Why are ENSDs assumed to be sourced from sulfate rather than reduced sulfur species that formed during the deposition and early diagenesis of the original evaporite deposits?
(3) Why is an oxidant for sulfide required if the ultimate sulfur source is sulfate, the most oxidized form of sulfur?
(4) Why is O_2_ considered the prime driver for sulfide oxidation in the canonical model?
(5) How could O_2_ be problematic for the formation of native sulfur if it is considered a prerequisite?
(6) Is there geochemical evidence from the rock record that indicates a lack of O_2_ supply to sites where epigenetic native sulfur formation took place?

### Direct Evidence for Involvement or Absence of O_2_ in the Genesis of ENSDs – The Example of Challenger Knoll

The fact that ENSDs form in the subsurface in hydrocarbon-bearing systems presents a major challenge to study the involved processes *in situ*. Epigenetic native sulfur deposits have been accessed by drilling and mining, but even in cases where native sulfur and hydrocarbons were present, such as at the Main Pass 299 dome offshore Louisiana (Kyle, 1999), it is not clear if the formation process was still ongoing. Moreover, drilling and mining introduce O_2_ and potentially cause contamination with extraneous microbial communities. For these reasons, interpretations of the conditions under which ENSDs formed mainly rely on petrographic or geochemical studies of geologic archives. Unfortunately, it is geochemically difficult to find evidence that O_2_ is involved in sulfide oxidation because this process does not leave obvious geochemical fingerprints. For example, during the oxidation of sulfide to native sulfur, oxygen from O_2_ reacts with hydrogen to form water, which is added to the pool of ambient water. The distinct isotopic fingerprint of the O_2_-derived water is diluted to such a degree that it is no longer distinguishable in the original ambient water, nor is it preserved in carbonate minerals that precipitate from this water. Moreover, chemotrophic sulfide-oxidizing bacteria do not synthesize lipid biomarkers of sufficient specificity (Arning et al., 2008). It is equally difficult to make a case, or find geochemical proof, that O_2_ was absent during the formation of an ENSD. Thus, other, more indirect approaches are necessary. For example, at Damon Mound it was shown that the hydrocarbon degradation products differ between the sulfur-barren near surface caprock, which has likely experienced extensive penetration of O_2_-rich meteoric water, and deeper caprock where native sulfur is present and supply with O_2_ must have been limited (Sassen et al., 1988).

To the best of our knowledge, Challenger Knoll, located in the Central Gulf of Mexico, is the only ENSD that has been accessed by scientific drilling and is a prime example of sulfur formation where little argument for the presence of O_2_ can be made. Challenger Knoll (DSDP Leg 1, Site 2) is a salt diapir that was drilled in 1968, in a water depth of 3,600 m (Ewing et al., 1969). Caprock was recovered from a depth of 133 m below sea floor (Burk et al., 1969). It was speculated that the intrusion of oxygenated seawater could be responsible for the presence of native sulfur (Davis and Bray, 1969). However, the pore water chloride profiles showed fluctuations that indicated salt dissolution (increase in chloride) as well as addition of water from the dissolution of gypsum and oxidation of hydrocarbons (decrease in chloride). No evidence for entrainment of seawater or diffusion of O_2_ through more than 100 m of pelagic sediment to the top of the zone with native sulfur was shown (Manheim and Sayles, 1970). From this, we conclude that the one site accessed to date with potentially active epigenetic native sulfur genesis has no evidence for presence of O_2_.

### Epigenetic Sulfur Deposits Are Truly Epigenetic

Evaporite-rich sedimentary rocks form in highly saline waters in arid continental settings or in ocean margin basins with high evaporation rates and limited water exchange with the global ocean. Such water bodies tend to become stratified due to density contrasts of highly saline bottom waters and fresher surface waters. Combined with the fact that O_2_ solubility decreases with increased salinity, such depositional settings are prone to become anoxic, which in addition to the precipitation of evaporite minerals, can lead to the deposition of organic carbon-rich sediments and to the formation of syngenetic native sulfur deposits through the activity of sulfate-reducing organisms (e.g., Philip et al., 1994; Aref, 1998; Ziegenbalg et al., 2010; Lindtke et al., 2011).

Based on these observations, the question arises: could ENSDs be formed from sulfur compounds with intermediate oxidation state and elemental sulfur that accumulated during the deposition and early diagenesis of the evaporites? While a contribution of these sources cannot be excluded, several lines of evidence indicate that they cannot replace reduction of sulfate from gypsum and anhydrite as the primary sulfur source due to the following reasons. (1) Syngenetic sulfur deposits are typically out-sized by bioepigenetic sulfur deposits, which accounted for more than 98% of the world sulfur production with one third being from caprock and almost two thirds from stratabound deposits (Ruckmick et al., 1979), indicating numerous syngenetic sulfur deposits would have to be recycled to obtain ENSDs. (2) Diagenetically formed tourmaline minerals in salt domes indicate that despite the fact that evaporite deposits can host organic-rich source rocks (e.g., the evaporitic Middle Pennsylvanian Paradox Formation in Utah and Colorado; Nuccio and Condon, 1996), the salt bodies themselves are likely not reduced (Henry et al., 1999; Henry and Dutrow, 2012). This confines potential accumulations of sulfur with intermediate oxidation states to these isolated bodies. (3) The typical paragenesis of carbonate and native sulfur minerals in epigenetic and syngenetic native sulfur deposits indicates that the two phases are precipitated in temporal proximity to each other, likely due to the coupling of sulfur and carbon cycling. If ENSDs were the result of a remobilization of sulfur compounds with intermediate oxidation state, a simultaneous formation may no longer be required – however, it is typically observed. (4) Carbonates in syngenetic native sulfur deposits tend to be ^18^O-enriched because the water from which the carbonates precipitate is isotopically heavy due to evaporation. In contrast, carbonates in ENSDs are typically ^18^O depleted because the associated brines are isotopically light. Thus, considering that there would not be enough sulfur compounds with intermediate oxidation state and that it is difficult to explain the late-stage paragenesis of native sulfur and carbonates as a result of remobilization, it follows that ENSDs are likely derived from sulfate minerals rather than reduced sulfur species generated in an early depositional environment.

### Canonically, Sulfide Is the Only Product of Dissimilatory Sulfate Reduction

**FIGURE 3:**
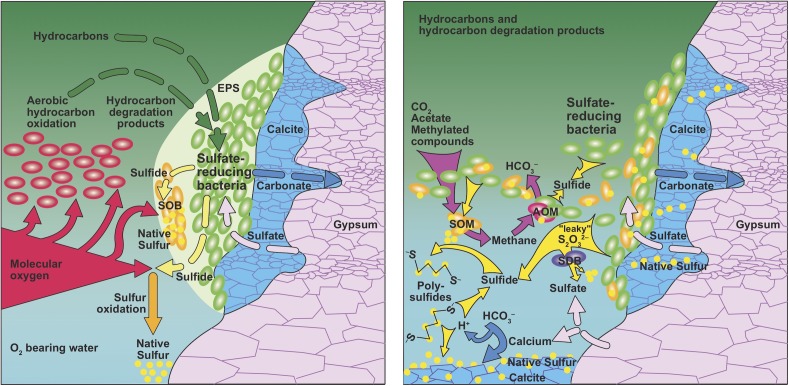
Genesis of native sulfur in presence (left) and absence (right) of O_2_. **(Left)** In a system where native sulfur genesis is driven by supply with O_2,_ there is a competion between oxygen-consuming aerobic hydrocarbon oxidation and oxidation of sulfide to native sulfur. Replacement of gypsum with carbonate is confined to the interface between solid gypsum and hydrocarbons in order to maintain low-oxygen conditions required for sulfate-reducing bacteria using extracellular polymeric substances (EPS). Sulfur-oxidizing bacteria (SOB) could help produce native sulfur in close association with sulfate-reducing bacteria. **(Right)** In an oxygen-free environment, sulfate reduction can take place detached from gypsum surfaces at the hydrocarbon-brine interface because gypsum dissolution provides sulfate to the brine. Sulfur cycling may include simultaneous genesis and consumption of methane and sulfate, constituting complete cryptic carbon and sulfur cycles. Sulfide-oxidizing microbes (SOM) produce methane and native sulfur. Sulfur disproportionating bacteria (SDB) convert sulfur compounds with intermediate oxidation state or native sulfur into sulfate and sulfide. Finally, anearobic oxidation of methane (AOM) consumes sulfate and methane. Sulfide can react with native sulfur to form polysulfides, a reaction that is reverted during carbonate precipitation, due to local increase in acidity.

There is a plethora of biological sulfur transformations, which includes sulfur reduction, oxidation, disproportionation and comproportionation reactions (e.g., Böttcher et al., 2005; Wasmund et al., 2017). Many of these reactions may create zero-valent sulfur as an intermediate or final product and it is likely that some of these processes have not been discovered yet, a fact that has been referred to as ‘cryptic sulfur cycling’ (Canfield et al., 2010b; Holmkvist et al., 2011; Johnston et al., 2014; Brunner et al., 2016). Microorganisms have been shown to use intermediate sulfur species, such as thiosulfate, to produce native sulfur (Figure 3). Examples are thermophilic mixolithoautotrophic bacteria growing on hydrogen and thiosulfate while producing native sulfur (Beffa et al., 1993) and the thermophile *Clostridium thermosulfurogenes*, which produces native sulfur from thiosulfate while fermenting carbohydrates to ethanol, molecular hydrogen, carbon dioxide, acetate, lactate, methanol, and isopropanol (Schink and Zeikus, 1983).

However, the source of new zero-valent sulfur must ultimately be the only large available sulfur pool in these deposits: sulfate from anhydrite or gypsum. Dissimilatory sulfate reduction is currently the only known energy-yielding microbial pathway capable of converting the stable sulfate molecule into a compound that can be reduced, adenosine phosphosulfate (APS). Sulfur compounds with intermediate oxidation state (e.g., sulfite, thiosulfate, trithionate, tetrathionate) are produced and released during oxidative sulfur cycling, but this does not appear to be the case for dissimilatory sulfate reduction which yields only one product: sulfide (for a review, see Barton et al., 2014). Sulfate reduction is also known for many assimilatory pathways (Schiff and Fankhauser, 1981), but this energetically costly sulfur transformation is not likely to be a good candidate as a supplier for sulfur compounds with intermediate oxidation state on a scale relevant for the genesis of large native sulfur deposits. It follows that while there is a multitude of processes that can yield native sulfur, canonically, dissimilatory sulfate reduction constitutes the only means to supply the system in which the ENSD is formed with new native sulfur. Because sulfide is presumably the only product of dissimilatory sulfate reduction, its subsequent oxidation is required. Theoretically, in a cryptic sulfur cycle such sulfur could be oxidized by a sulfur compound with intermediate oxidation state, the latter thereby becoming more reduced. However, unless there is an external oxidant such as O_2_ that re-constitutes sulfur compounds with intermediate oxidation states, this cryptic cycle cannot be perpetuated because oxidation power is progressively lost.

### Molecular Oxygen as an Agent for the Genesis of ENSDs

Evaporite deposits dominantly consist of carbonates, sulfates, and sodium chloride. Iron or manganese are rare constituents, as are nitrate salts due to their high solubility. From this follows that other than sulfate, evaporite rocks contain few compounds that could serve as oxidants for hydrocarbons or sulfide. Until the late 1980s, little evidence was available that anaerobic microorganisms degraded hydrocarbons. It was well established that aerobic degradation of hydrocarbons by O_2_, acting as a strong oxidant, was directly involved in overcoming the chemical sluggishness of hydrocarbon oxidation (Widdel and Rabus, 2001). Combined with the knowledge that sulfate-reducing bacteria mostly depend on products of hydrocarbon degradation (Jobson et al., 1979), such as organic acids, this information led to the conclusion that “sulfate reducers are anaerobes but typically depend on aerobic bacteria to create suitably biodegraded hydrocarbon substrates” (Figure 3; Warren, 2006) and that meteoric water supplies the bulk of dissolved oxygen, whereas formation fluids supply oil or methane for the genesis of calcitic caprocks on salt diapirs (Kyle and Posey, 1991). Thus, since it was accepted that O_2_ needed to be available for the degradation of oil, it was conceivable that O_2_ would also be available for the oxidation of sulfide to native sulfur, while acknowledging that sulfate-reducing bacteria, which produce the sulfide, are strict anaerobes (Machel, 1992).

Yet, since the late 1980s, the concept that only aerobic microorganisms can decompose hydrocarbons has dramatically changed (Head et al., 2003; Aitken et al., 2004; Gray et al., 2010). An increasing number of microorganisms were identified that have the ability to utilize saturated and aromatic hydrocarbons as growth substrates under strictly anoxic conditions, and sulfate-reducing bacteria were shown to use a much broader range of substrates from oil and gas reservoirs than previously assumed (Aeckersberg et al., 1991; Fukui et al., 1999; Widdel and Rabus, 2001; Rabus et al., 2006; Widdel et al., 2006, 2010; Kniemeyer et al., 2007). The recent finding that butane-oxidizing archaea function in a consortium with sulfate-reducing bacteria further underlines that anaerobic hydrocarbon oxidation coupled to sulfate reduction can take advantage of a broad range of substrates (Laso-Pérez et al., 2016). These findings do not only apply to incubation studies but are corroborated by biomarker studies. Biomarkers that are attributed to anaerobic methanotrophic archaea (ANME) and associated sulfate-reducing bacteria, who carry out sulfate-driven anaerobic oxidation of methane (AOM), were found in carbonates associated with native sulfur deposits in Sicily (Ziegenbalg et al., 2012). Similarly, biomarker evidence for a microbial community dominated by sulfate-reducing bacteria that is capable of using petroleum hydrocarbons to reduce gypsum and produce carbonates was found in diagenetic carbonates associated with native sulfur deposits from the Gulf of Suez (Aloisi et al., 2013). These findings led to the conclusion that O_2_ is not needed for the coupling of oil and gas degradation to microbial sulfate reduction, and by extension, imply that O_2_ may not be available for the oxidation of sulfide to native sulfur.

### Tolerance of Sulfate-Reducing Microbes to O_2_, Microniches to Separate Sulfur Oxidation From Reduction and Competition for O_2_ in Hydrocarbon Oxidation

Most sulfate-reducing bacteria function anaerobically but do have the ability to tolerate O_2_ as a means for survival (Cypionka et al., 1985; Marschall et al., 1993; Fauque, 1995; Cypionka, 2000; Rabus et al., 2006; Ramel et al., 2015). Molecular oxygen is not toxic to sulfate-reducing bacteria, but the reaction of O_2_ with reduced sulfur compounds releases toxic products, such as thiols, which may explain why O_2_ appears to be more toxic to metabolizing than resting cells (Cypionka, 2000). The substrates for aerobic respiration by sulfate-reducing bacteria are the same as the substrates used in sulfate reduction. It has been shown that when exposed to O_2_, the metabolically versatile sulfate-reducing bacterium *Desulfobulbus propionicus* can generate native sulfur as an intermediate (Fuseler and Cypionka, 1995). However, despite (1) their capacity to couple O_2_ reduction with energy conservation, (2) their chemotaxis toward micro-aerobic zones, and (3) their detoxification mechanisms, proof has yet to be provided for aerobic growth of sulfate-reducing bacteria, i.e., over an infinite number of generations in oxic media (Rabus et al., 2006). In environmental samples, including a supralitoral marine microbial mat (Visscher et al., 1992), hypersaline microbial mats (Canfield and Marais, 1991; Jørgensen, 1994), root zones (Isaksen and Finster, 1996; Blaabjerg and Finster, 1998), and biofilms from a sewage plant (Kühl and Jørgensen, 1992), it has been shown that sulfate reduction can take place under oxic conditions. So far, sulfide production in pure cultures of sulfate-reducing bacteria in the presence of O_2_ has not been documented (Cypionka, 2000). Recent discoveries indicate that there is the possibility of sulfate-reducing microorganisms growing under oxic conditions. The diversity of microbial groups that may be involved in dissimilatory sulfur cycling is much larger than previously thought (Rückert, 2016; Anantharaman et al., 2018). Among these microorganisms are peatland *Acidobacteria* that possess the genomic toolset to convert sulfite into sulfide with a subgroup that also possesses the toolset to convert sulfate into sulfite (Hausmann et al., 2018). This is of particular interest because cultivated *Acidobacteria* related to the peatland *Acidobacteria* are aerobes or facultative anaerobes (Hausmann et al., 2018 and references therein). However, since O_2_ is energetically a much more attractive electron acceptor than sulfate, it remains uncertain why dissimilatory sulfate reduction would be carried out if O_2_ is persistently available, and why such organisms would not be out-competed by aerobic competitors. Moreover, persistent presence of O_2_ would keep sulfide concentrations low. Sulfide inhibits native sulfur disproportionation (Thamdrup, 1993; Finster et al., 1998). Removing this inhibitor would enable a process that consumes native sulfur, and thus not allow for the formation of a native sulfur deposit.

This gives rise to the question of how the supply with O_2_ can be regulated such that the sulfate-reducing microbes are not negatively affected? In shallow water including reefal environments, sulfate-reducing organisms can thrive within millimeters of an oxic-anoxic interface (Visscher et al., 2000; Dupraz et al., 2004; Fike et al., 2008), and biomarker and isotopic fingerprints of this process, occurring in presumably oxic environments with only anoxic micro-niches present, can be preserved in the geologic record (Heindel et al., 2012; Gischler et al., 2017a,b). Likely, the steep chemical gradients are maintained by extracellular polymeric substances, for example in biofilms, which limit the exchange of O_2_ between oxidizing and reducing microenvironments. However, formation of biofilms may not be favored in epigenetic settings due to lack of pore space and no fossilized biofilms are reported from these subsurface environments. Lacking the protection of biofilms, sulfate-reducing bacteria can be exposed to O_2_ more easily.

Sulfide diffusing toward an oxidizing environment can be oxidized to native sulfur if hydrogen sulfide has a long enough residence time before being oxidized by other processes (Figure 3). In stratabound or salt diapir settings, such environments could potentially exist at locations with crossflow of oxygenated water adjacent to a dissolving salt, gypsum, or anhydrite (Figure 1; Warren, 2006). The locally increased salinity reduces the solubility of O_2_, facilitating the establishment of anoxic microniches. For this model to function, the supply of hydrocarbons or products of hydrocarbon degradation to the sulfate reducers must be maintained (Figure 3). Anaerobic microorganisms utilizing hydrocarbons always exhibit much slower growth than their aerobic counterparts (Widdel et al., 2010), and sulfate-reducing bacteria are outcompeted by aerobes, as exemplified by the classical redox sequence in marine sediments (Canfield and Thamdrup, 2009). This leads to a conundrum: hydrocarbons must be delivered to the sulfate-reduction zone without exhausting the O_2_ in the oxygenated water since O_2_ is needed for the subsequent oxidation of sulfide to native sulfur, or sulfide oxidation to native sulfur with O_2_ must be kinetically faster than aerobic oxidation of hydrocarbons (Figure 3). The former option appears to be unlikely because if excess O_2_ was present, sulfur oxidation would likely proceed all the way to sulfate. The latter scenario might be possible, as sulfide is a highly effective antioxidant. However, to accumulate a large native sulfur deposit *in situ*, supply with oxygenated water needs to be large enough to maintain sulfide oxidation, despite the competition for O_2_ by aerobic hydrocarbon degradation. Due to the density stratification with gas and oil on top of water, such a massive inflow of O_2_ must come from a lateral source (Figure 1).

An alternative to the above scenario is the spatial or temporal separation of microbial sulfate reduction from sulfide oxidation. Sulfide is allowed to accumulate *in situ* during a period where hydrocarbons are available for sulfate reduction with no O_2_ supply, followed by a phase where O_2_ is present but no hydrocarbons are available, allowing sulfide to be oxidized to native sulfur. Such a separation of sulfate reduction and sulfide oxidation can also be achieved by migration of sulfide to a location where O_2_ is available. The latter scenario requires a stratified water body, whereas the former relies on episodic changes in fluid supply (Jassim et al., 1999), which may be tied to shifts in the groundwater table due to seasonal changes, or in coastal areas, due to sea level fluctuations or changes in seepage of oil into the caprock system, triggering changes in the location of the interface between groundwater and captured hydrocarbons (Figure 1). One implication of these scenarios is that carbonate precipitation, which is tied to hydrocarbon oxidation, and native sulfur accumulation, can be temporally or spatially separate.

The discussion of (1) the O_2_-tolerance of sulfate-reducing microbes, (2) microniches to separate sulfur oxidation from reduction, and (3) competition for O_2_ in hydrocarbon oxidation demonstrates that presence of O_2_ does not *a priori* exclude the formation of native sulfur and that it is likely such systems do exist. However, it shows that if supply with O_2_ is critical for the genesis of ENSDs, favorable circumstances need to coincide as both excess or dearth in O_2_ can be problematic.

### Assessment of Fluid Flow: Geologic Evidence for Native Sulfur Formation in Absence of O_2_

Whereas overabundance of O_2_ could impede sulfide generation and lead to the oxidation of native sulfur, insufficient supply with O_2_ may result in the escape of sulfide and absence of native sulfur. Molecular oxygen in the subsurface can move by diffusion or advection, but because O_2_ diffusion on a 100m to km scale is exceedingly slow (e.g., Røy et al., 2012), advective transport is essential. Sustained fluid flow through the rocks adjacent to the native sulfur body can be driven by topographic differences (Figure 1), dewatering of clay minerals or gypsum at depth, or density gradients caused by differences in salinity and/or temperature resulting in the establishment of convection cells (Hanor, 1994; Thornton and Wilson, 2007). The fluids can range from meteoric to highly saline basinal brines (Hanor, 1994; Kharaka and Hanor, 2014). Systems in which ENSDs form cannot be closed to fluid exchange since there must be a supply of hydrocarbons and, in the case of salt diapirs, salt must be removed via dissolution. If O_2_ is supplied continuously or episodically, these fluxes must contribute to the transport of other solutes as well. Therefore, carbon, oxygen, and sulfur isotope mass balances have the potential to reveal if the flow of fluids was ample or limited.

#### Carbon and Oxygen Isotope Composition of Carbonates, Water, and Carbon Sources

**FIGURE 4:**
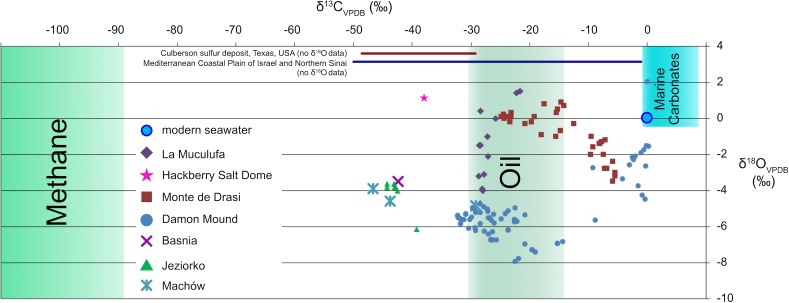
Carbon isotopes systematics in epigenetic sulfur deposits. Compilation of carbon isotope data from carbonates from epigenetic native sulfur deposits. Thermogenic or biogenic methane is isotopically very light and may have contributed to the formation of carbonates at various sites, such as the south-eastern Mediterranean Coastal Plain of Israel and Northern Sinai (Nissenbaum, 1984), the Hackberry salt dome, Louisiana, United States (McManus and Hanor, 1993); the Carpathian Foredeep, Poland (Parafiniuk et al., 1994; Böttcher and Parafiniuk, 1998), Sicily, Italy (Ziegenbalg et al., 2012), and within stratiform native sulfur deposits in the Castile anhydrite in the northwestern and west-central Delaware Basin, United States (Kirkland, 2014). The carbon isotope composition of carbonates from Damon Mound (this study) fall almost entirely into the range between oil-derived carbon and carbonate from seawater, but a contribution from methane cannot be excluded.

The oxygen isotope composition (δ^18^O) of carbonates can provide information about the fluids from which they precipitated, whereas their carbon isotope composition (δ^13^C) provides information about the carbon source. For example, a δ^18^O value of 0.05‰ and a δ^13^C value of −25.30‰ (vs. PDB-I; Davis and Bray, 1969) for calcite from the Challenger Knoll caprock indicate precipitation from fluid with a near-seawater oxygen isotope composition (Davis and Bray, 1969). The carbon isotope value indicates that oil was the dominant carbon source, as it is very similar to oil found in Challenger Knoll (Davis and Kirkland, 1979). Carbon isotope values lower than −30‰ indicate contribution of carbon from thermogenic or biogenic methane (Schoell, 1980), and have been reported for native sulfur deposits in the south-eastern Mediterranean Coastal Plain of Israel and Northern Sinai (Nissenbaum, 1984), the Hackberry salt dome, Louisiana, United States (McManus and Hanor, 1988), the Carpathian Foredeep, Poland (Parafiniuk et al., 1994; Böttcher and Parafiniuk, 1998), Sicily, Italy (Ziegenbalg et al., 2010), and within stratiform ENSDs in the Castile anhydrite in the Delaware Basin, United States (Kirkland, 2014). The three-component system with marine dissolved inorganic carbon (δ^13^C close to 0‰), oil (δ^13^C as low as −30‰), and methane (δ^13^C as low as approximately −100‰), makes it difficult to establish a precise mass balance between the different pools and to quantify the fluxes involved (Figure 4). Still, low δ^13^C values indicate limited supply of oxygenated water, as such waters would contribute isotopically heavy carbon.

#### Sulfur Isotopes in ENSDs

**FIGURE 5:**
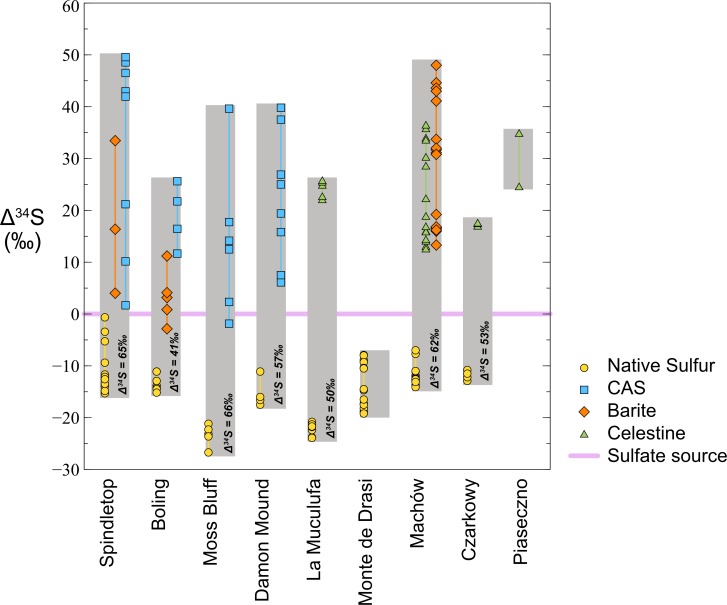
Sulfur isotopes systematics in epigenetic sulfur deposits. Compilation of data from United States Gulf Coast, Polish, and Sicilian native sulfur deposits, data normalized to presumed sulfur isotope composition of sulfate source (set to zero, data presented as an offset between isotope compositions; Δ^34^S). The sulfur isotope compositon of native sulfur is lighter and the residual sulfates isotopically heavier than the original sulfate mineral. The isotope offset between native sulfur and original sulfate is much smaller than the offset between original sulfate and residual sulfate. However, the enrichment in ^34^S does not exceed the theoretical maximum isotope fractionation for microbial sulfate reduction. *Data sources:* Huckley, Boling, Moss Bluff, and Spindletop domes (Feely and Kulp, 1957; Kyle and Agee, 1988); Damon Mound (new data); Poland (Parafiniuk et al., 1994); Sicily (Ziegenbalg et al., 2010).

In contrast to the three carbon sources, there is typically only one major sulfur source in ENSDs – sulfate from evaporite minerals. These sulfates tend to have a fairly uniform sulfur isotope composition (δ^34^S) because they were formed from a large seawater sulfate pool and precipitation of sulfate minerals is associated with a small isotope fractionation (Thode and Monster, 1965; Holser and Kaplan, 1966; Lloyd, 1968; Pirlet et al., 2010; Pichat et al., 2017). The components of the sulfur cycle fall into five basic categories: (1) original sulfate in gypsum or anhydrite, (2) residual sulfate that has been exposed to microbial sulfate reduction, (3) sulfide minerals, (4) sulfurized organic compounds, and (5) native sulfur. In the absence of suitable cations, such as iron, no sulfide minerals (i.e., pyrite) are formed. If sulfurization of oil from its reaction with hydrogen sulfide can be ignored, the system reduces to three main categories. Residual sulfate can precipitate in secondary minerals: anhydrite or gypsum, celestine (SrSO_4_), barite (BaSO_4_) or as carbonate associated sulfate (CAS) in authigenic carbonates (Figure 2). The sulfur isotope systematics of native sulfur deposits show elemental sulfur is isotopically lighter and the residual sulfates isotopically heavier than the original evaporite minerals. Typically, the range in δ^34^S of the native sulfur is smaller than that of residual sulfates, and the difference in isotope composition between native sulfur and the original sulfate is much smaller than that between original and residual sulfate (Figure 5). Classically, such signatures have been interpreted as the result of microbial sulfate reduction in a closed system. Sulfate-reducing bacteria preferentially produce sulfide that is depleted in ^34^S relative to sulfate, thereby enriching the residual sulfate in ^34^S. With continuing consumption of the remaining sulfate, the δ^34^S value of residual sulfate becomes exponentially heavier, whereas the accumulated sulfide approaches the δ^34^S value of the original sulfate, a process that can be modeled as closed system Rayleigh distillation (Faure, 1986; Hoefs, 2008). If small to moderate sulfur isotope fractionations in a range of 15‰ (Thode and Monster, 1965) to 27‰ (Feely and Kulp, 1957) are attributed to dissimilatory sulfate reduction, these trends match the observations of the sulfur isotope patterns of ENSDs fairly well (Figure 5). This led to the conclusion that the inhomogeneous, heavy isotope signatures of sulfate remaining in the calcite cap rock represents residues from bacterial sulfate reduction (Feely and Kulp, 1957).

#### Closed vs. Open System Sulfur Isotope Fractionation

There are several reasons why a Rayleigh distillation model may not be appropriate to interpret data from ENSDs. The first is that in proximity to massive gypsum or anhydrite deposits, there is always ample supply of sulfate, thus the system is not closed with respect to sulfate input. Second, if hydrocarbons, and potentially oxygenated water enter the system, the fluids they replace would be expected to entrain produced sulfide and residual sulfate, once more making a closed system argument difficult to uphold. Last, for a closed-system Rayleigh isotope fractionation, the maximum sulfur isotope offsets observed between original and residual sulfate as well as native sulfur appears to be too small. Isotope fractionation between sulfate and sulfide during sulfate reduction can be as large as 75‰, particularly if the overall energy yield for the sulfate-reducing organisms is low, as it is likely for oil-derived compounds (Rudnicki et al., 2001; Wortmann et al., 2001; Kleikemper et al., 2004; Brunner and Bernasconi, 2005; Canfield et al., 2010a; Sim et al., 2011a,b; Wing and Halevy, 2014). The same holds for sulfate reduction associated with AOM, particularly if methane supply is low (Deusner et al., 2014). This begs the question, why, at least for some ENSDs, is the sulfur isotope offset between native sulfur and the original sulfate not much larger for an initial stage of Rayleigh distillation, and also much larger between original and residual sulfate at a late stage? If one excludes thermochemical sulfate reduction at low temperatures (70°C) as a means to explain the minimal isotope offset between sulfide and original sulfate, which was an option proposed for West Huckberry dome (McManus and Hanor, 1993), an answer must be found that accommodates the observed moderate degree of sulfur isotope fractionation, continuous supply of sulfate from gypsum or anhydrite dissolution, and some degree of transport of solutes out of the system.

#### Implications From Open System Sulfur Isotope Fractionation

**FIGURE 6:**
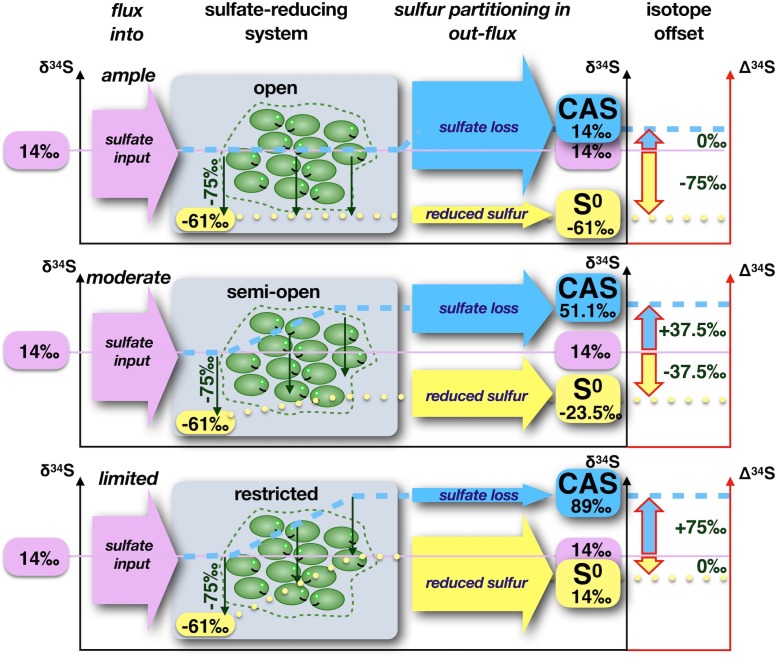
Systems with ample and restricted fluid flow (i.e., removal of sulfate). Examples for sulfur isotope patterns in a system with ample, semi-restricted and restricted fluid flow. The input of sulfate from dissolution of gypsum/anhydrite (chosen δ^34^S of +14‰), the sulfur isotope fractionation by sulfate reduction is assumed to be 75‰. **(Top)** If most of the sulfate that enters the system also leaves the system (ample fluid flow), the δ^34^S of the residual sulfate (CAS) matches the δ^34^S of original sulfate and the δ^34^S of native sulfur is strongly offset to low values. **(Middle)** If half of the sulfate entering the system is converted to native sulfur, native sulfur and residual sulfate will have the same absolute isotopic offset from residual sulfate. **(Bottom)** If essentially all sulfate that enters the system is converted to native sulfur, the δ^34^S of native sulfur matches the δ^34^S of original sulfate, and the δ^34^S of the residual sulfate is strongly offset to isotopically heavier values.

In treating the formation of ENSDs as an open or semi-open system, three fluxes are considered to operate in a quasi-steady state: the input of sulfate from dissolution is matched by the precipitation of native sulfur and the removal of residual sulfate by transport out of the system, by precipitation as a sulfate mineral, or by capture as CAS. Similarly, there must be a match between the isotope composition of sulfur that enters the system as sulfate and the sulfur that leaves the system and precipitated native sulfur, whereby the latter two are offset by the sulfur isotope fractionation (Figure 6). If most of the sulfate that enters the system also leaves, the δ^34^S value of the residual sulfate matches the δ^34^S value of original sulfate and the δ^34^S value of native sulfur is strongly offset to low values. If essentially all sulfate that enters the system is converted to native sulfur, the δ^34^S value of native sulfur matches the δ^34^S value of original sulfate, and the δ^34^S value of the residual sulfate is strongly offset to isotopically heavy values. If half of the sulfate entering the system is converted to native sulfur, native sulfur and residual sulfate will have the same absolute isotopic offset from residual sulfate. It is important to note that in such an open system, the isotopic offset between native sulfur and residual sulfur is approximately equal to the sulfur isotope fractionation by sulfate-reducing organisms, which should have a maximum of approximately 75‰ (e.g., Brunner and Bernasconi, 2005; Sim et al., 2011a). In the closed, Rayleigh-type system the difference could be much larger because residual sulfate becomes exponentially enriched in ^34^S, while accumulated sulfide and native sulfur approach the δ^34^S value of original sulfate. Remarkably, for the available data sets, the spread between residual sulfate and native sulfur remains within the 75‰ range (Figure 5). In this view, it becomes evident that in the majority of the studied native sulfur deposits, sulfate conversion to native sulfur exceeded the loss of sulfate, which implies that fluid transport in and out of the system during sulfide generation must have been restricted. In cases where the δ^34^S value of native sulfur approaches the value of original sulfate, external fluid input must have been almost cut off. Such a scenario is not compatible with concomitant supply of oxygenated water.

It can be argued that celestine and barite only form during late stage sulfate reduction in an essentially closed system when enough barium and strontium have built up but sulfate has not been consumed to a degree where precipitation can no longer occur. Then their δ^34^S values would only record a specific segment of the isotope trends, which could be taken as an argument why the difference between the residual sulfate and native sulfur remains limited to the 75‰ range. For this reason, analysis of the δ^34^S values of CAS may be more appropriate, because authigenic carbonates are expected to keep precipitating as long as carbonate production through hydrocarbon oxidation proceeds. A compilation of values from ENSDs from the United States Gulf coast, Poland, and Sicily, Italy shows that the sulfur isotope offset between native sulfur and CAS, barite, and celestine is well within the 75‰ range (Figure 5). For example, at Damon Mound, the highest δ^34^S value of CAS is 55.8‰, and the lowest δ^34^S of native sulfur is −1.5‰, which results in an offset of 57.3‰ (Figure 5 and Table 1). Using this offset as our sulfur isotope fractionation value, applying it to the highest measured δ^34^S value of native sulfur of 4.9‰, and assuming a value of 16‰ for gypsum from Louann Salt formation that is the likely sulfate source (Feely and Kulp, 1957; Claypool et al., 1980; Kyle and Agee, 1988; Prikryl et al., 1988), we find that more than 80% of the sulfate was converted to native sulfur and less than 20% of residual sulfate was removed, either into a solid phase or as dissolved compound. Thus, at Damon Mound fluid flow must, at least intermittently, have been sluggish. Accumulation of sulfide, due to the lack of O_2_ supply would have been a likely consequence.

**Table 1:** Data from Damon Mound.

Sample label	Sample description	Phase	δ^13^C (‰)	δ^18^O (‰)	δ^34^S (‰)
DMCCR-001a	Light gray	CAS	−22.2	−5.7	22.1
DMCCR-001c	Darker gray	CAS	−22.6	−5.6	23.5
DMCCR-013a	White crystallized calcite vein	CAS	−23.6	−6.2	55.8
DMCCR-013b	Regular light gray matrix	CAS	−32.3	−5.4	41.0
DMCCR-013c	darker gray laminated ’blob’	CAS	−31.8	−5.9	53.5
DMCCR-015a	Gray matrix	CAS	−15.3	−6.9	43.0
DMCCR-018a	Light gray section	CAS	−29.7	−5.0	35.4
DMCCR-018b	Light/dark mixed zone	CAS	−27.2	−5.0	31.8
DMCCR_1a_S0	Native sulfur	S^0^	–	–	−17.5
DMCCR_1b_S0	Native sulfur	S^0^	–	–	−16.6
DMCCR_2a_S0	Native sulfur	S^0^	–	–	−16.0
DMCCR_2b_S0	Native sulfur	S^0^	–	–	−11.1

In summary, the assessment of carbon, oxygen, and sulfur isotope mass balances for ENSDs demonstrates that fluid flow must have been sluggish. This implies that supply with O_2_ must have been insufficient for the genesis of the native sulfur at these sites, and that conditions at the time of the formation of native sulfur were probably hypersulfidic. In addition, the carbon isotope signature of the carbonates shows that oil and methane have served as electron donors for sulfate reduction, implying that there is diversity in the microbial communities that carry out the overall process.

## Alternatives to the Oxidation of Sulfide With O_2_ for the Genesis of Epigenetic Native Sulfur Deposits

The discussion above shows that if O_2_ were the oxidant in native sulfur deposits, several biogeochemical coincidences are required. For example, well-timed alternating cycles of abundant O_2_ with phases of low supply, where the sulfide can accumulate, have been postulated for the Middle Miocene strata-bound sulfur deposits of northern Iraq (Jassim et al., 1999), or a ‘pipe-to-chimney-like’ saline groundwater flow into the Castile evaporite in the Rustler Springs sulfur district, Texas (Kirkland, 2014). Moreover, geochemical data imply that supply of O_2_ to the sites where native sulfur deposits were formed was limited, and that for Challenger Knoll – the rare case where a site with potentially active sulfur generation was accessed – O_2_ was found to be absent. While explanations that include O_2_ as critical agent in the formation of native sulfur deposits may be plausible for some sites, following the law of parsimony (Occam’s razor), we pose the question: are there not simpler – and less coincidental – alternatives for the genesis of native sulfur deposits?

Under the presumption that the sulfur in ENSDs is ultimately derived from sulfate, a microbiological answer to this geological conundrum could involve several concepts. One is an alternative, or ‘leaky,’ dissimilatory sulfate reduction pathway that yields sulfur compounds with intermediate oxidation states, which can subsequently be converted to native sulfur. Another could utilize ‘non-classical’ oxidants and oxidation pathways for the oxidation of sulfide to native sulfur. Any postulated scenario has to be energetically feasible. If the proposed process argues for a shift from one metabolic pathway to another, there should be a reason for what could have triggered such a shift. Ideally, there should also be evidence from experiments or the environment that point to the possible existence of the hypothesized process.

In the following, we will explore these points by addressing three questions:

(1) Has native sulfur formation with sulfate as the ultimate sulfur source been observed in the absence of classical oxidants?
(2) What could cause a shift in sulfur metabolism, particularly for cases where this results in a lower energy yield?
(3) Are the hypothesized processes thermodynamically and kinetically feasible?

### Evidence for Native Sulfur Formation in the Absence of a Classical Oxidant

Direct evidence for the formation of native sulfur in the absence of a classical oxidant or light is scarce. The simplest explanation for this would be that such processes do not exist. Alternatively, it is likely that native sulfur formation in the absence of a classical oxidant would not be detected, as other sulfur cycling processes would obscure it. For example, neo-formed native sulfur could be removed or obscured by the addition of native sulfur that is derived from sulfide oxidation by a classical oxidant. The phenomenon that hidden processes in the sulfur cycle are difficult to detect is not uncommon and has been referred to as cryptic sulfur cycling (Canfield et al., 2010b; Holmkvist et al., 2011; Johnston et al., 2014; Brunner et al., 2016; Wasmund et al., 2017). Despite these constraints, there are two instances in which there is indication that native sulfur formation takes place in the absence of a classical oxidant, the anaerobic oxidation of methane coupled to sulfate reduction (AOM), and sulfur cycling in the sulfidic, hypersaline Urania deep-sea basin.

#### Native Sulfur Observed in Microbial Cultures: The Curious Case of AOM

For AOM, zero-valent sulfur was detected within ANME cells, the methane-oxidizing archaeal partners of sulfate-reducing bacteria (Milucka et al., 2012). The authors of the study hypothesized that the studied ANME, which belong to the ANME-2 cluster, might reduce sulfate to zero-valent sulfur, which is then disproportionated to sulfate and sulfide by bacterial partners, implying that ANME, who lack key enzymes of the classical dissimilatory sulfate reduction pathway (Timmers et al., 2017) use a different sulfate activation mechanism (Milucka et al., 2012, 2013). It has been shown that methane oxidation by ANME can be decoupled from sulfate reduction (Scheller et al., 2016), indicating that ANME are not responsible for sulfate reduction. Nevertheless, the observation that zero-valent sulfur was detected within ANME cells remains valid and is further corroborated by the finding of zero-valent sulfur with AOM biomass in a two stage high-pressure continuous incubation experimental system (Deusner et al., 2014). Bacteria that disproportionate native sulfur have been identified in sediment-free, long-term AOM enrichments of cultures that were obtained from coastal hydrocarbon seeps from Elba Island, Italy, from hot vents of the Guaymas Basin, Gulf of California, and methane seeps in the vicinity of the Gullfaks oil field in the North Sea (Wegener et al., 2016). These sulfur-disproportionating bacteria likely persisted because native sulfur was available, either due to oxidation of sulfide by O_2_ introduced during cultivation, or from the ANME (Wegener et al., 2016). Sulfur-disproportionating bacteria also appear to be present in oxygen-free environments, such as cold seeps, with active AOM (e.g., Lloyd et al., 2006; Niemann et al., 2009; Orcutt et al., 2010; Ruff et al., 2015) with unknown sources of native sulfur. These findings are intriguing since AOM most likely plays a role in the formation of a suite of native sulfur deposits, evidenced by (1) the very light carbon isotope signatures of authigenic carbonates (Figure 4) and (2) presence of archaeal lipids in native sulfur containing limestones from Sicily that are similar – but not identical – to lipids of ANMEs from marine methane seeps (Ziegenbalg et al., 2012).

#### Apparent Genesis of Sulfur Compounds With Intermediate Oxidation State in the Highly Sulfidic, Hypersaline Urania Deep-Sea Basin

Somewhat more circumstantial evidence comes from the Urania deep-sea basin. In the most saline layers of the basin, methanogenesis greatly exceeds sulfate reduction, pointing to methylated compounds, which cannot be utilized by sulfate-reducing bacteria as a substrate (Borin et al., 2009). However, neither a change in the ratio of bacteria to archaea nor a decrease in the relative abundance of *Deltaproteobacteria*, to which sulfate-reducing bacteria belong, was observed, causing the authors to conclude that the *Deltaproteobacteria* “are mainly performing functions other than sulfate reduction, possibly by using electron acceptors such as thiosulfate, sulfur, or dimethyl sulfoxide” (Borin et al., 2009). Unless these compounds with intermediate oxidation state are derived from oxidative sulfur cycling at the interface to oxic water, there would need to be a process within the sulfidic water body that supplies them. The co-occurrence of methanogenesis and native sulfur reduction – and simultaneous competition between these processes – in methanogens implies that there are close evolutionary relationships between these pathways (Stetter and Gaag, 1983), which could be exploited in the coupling of methanogenesis and native sulfur production. Thus, the question becomes: what could trigger the formation of zero-valent sulfur in hypersaline, sulfidic environments, and is there a link to carbon cycling via methanogenesis?

### Shifts in Sulfur Metabolism: Native Sulfur Genesis as a Stress Response

There are several reasons why metabolic processes can yield a product that is commonly absent. One cause is a change in the environmental conditions that results in an inactive process becoming more energy yielding than the previously prevailing one. A second option is a response to environmental stress, which may cause a metabolic process to no longer function optimally (but still energetically favorably), thereby releasing intermediates from its pathway. A third option constitutes a trade-off situation: to cope with unfavorable environmental conditions, organisms shift from a mechanism that overall provides the highest energy yield, but also leads to poor environmental conditions to a process with lower energy yield that offers the advantage that the reaction products do not exacerbate the unfavorable conditions.

Release of intermediates as a stress response has indeed been observed for dissimilatory sulfate reduction. This process operates over several steps, including intermediates such as adenosine phosphosulfate (APS), and sulfite (Akagi, 1995). Sulfite is reduced by the enzyme DsrAB, with the sulfite-derived sulfur coupling to DsrC, forming a protein-based trisulfide, which can then be reduced to reduced DsrC and sulfide (Santos et al., 2015). This last step is of particular importance for dissimilatory sulfate reduction since it couples the four-electron reduction of DsrC to energy conservation (Santos et al., 2015). In absence of functional DsrC but presence of DsrAB, the products of sulfate reduction can be trithionate, thiosulfate, and sulfide, however, DsrC turns out to be essential for sulfate reduction (Santos et al., 2015). Probably, the energy gained from this last step ties into the energetically costly activation of the sulfate molecule to APS, which could explain why dissimilatory sulfate reduction usually does not release sulfur intermediates. Nevertheless, it is known that under stressed conditions, such as maintenance metabolism in a retenostat or sulfite reduction by washed cells, thiosulfate and tetrathionate can be released by sulfate-reducing bacteria (Fitz and Cypionka, 1990; Davidson et al., 2009). Such a leakiness of dissimilatory sulfate reduction as a stress response to sulfate-saturated pore fluids coupled with low availability of an electron donor has been proposed to explain the formation of native sulfur nodules in the Lisan Formation, Israel (Bishop et al., 2013).

In the formation of ENSDs – particularly if they function as systems with rather sluggish fluid exchange – there are two obvious stress factors. The first factor is build-up of high levels of sulfide (sulfide stress). Following Le Chatelier’s principle this renders additional sulfide production energetically less favorable, but more importantly, at high levels, sulfide becomes toxic to organisms. Because ENSDs form in the vicinity of evaporite deposits, which offer vast quantities of highly soluble salts, the second stress factor is high concentrations of ions (salt stress).

#### Sulfide Stress

In aqueous solutions at circum-neutral pH, sulfide exists in approximately equal amounts as dissolved hydrogen sulfide (H_2_S) and bisulfide ion (HS^−^). In natural environments, sulfide concentrations can reach hypersulfidic levels, such as 10 mM in cool-water carbonate sediments, (Wortmann et al., 2001) or 16 mM in hypersaline waters of the Urania Deep (van der Wielen et al., 2005). Sulfide anions are as toxic as cyanide because they share the ability to coordinate and precipitate metal cations that are crucial for metabolism (for a review, see Beauchamp et al., 1984). A contributing factor to the toxicity of sulfide is the ability of the neutrally charged H_2_S to easily diffuse through cell membranes without facilitation of membrane channels (Barton et al., 2014). Sulfide is also toxic to the sulfate-reducing bacteria themselves. Most can tolerate sulfide concentrations of 10 mM and higher. However, sulfide stress results in community shifts, and lower growth and sulfate reduction rates (Widdel, 1988; Oleszkiewicz et al., 1989; McCartney and Oleszkiewicz, 1991; Okabe et al., 1992, 1995; Reis et al., 1992;Maillacheruvu and Parkin, 1996; O’Flaherty et al., 1998; Icgen and Harrison, 2006; Caffrey and Voordouw, 2010; Eckert et al., 2011). High sulfide concentrations are likely to increase the reversibility of the dissimilatory sulfate reduction pathway, which is expressed as increase in the observed sulfur isotope fractionation (Brunner and Bernasconi, 2005; Eckert et al., 2011). In sulfide-stress experiments with *Desulfovibrio vulgaris* Hildenborough, genes involved in energy production and conservation were found to be mostly downregulated under high sulfide conditions, with *dsrD* being the most affected gene of the entire genome (Caffrey and Voordouw, 2010). It has been proposed that the DsrD protein plays a role in transcription or translation of genes for enzymes catalyzing dissimilatory sulfite reduction (Mizuno et al., 2003), which resonates with the finding that in absence of functional DsrC but presence of DsrAB, the products of sulfate reduction can be trithionate, thiosulfate, and sulfide (Santos et al., 2015). Overall, these findings imply that sulfate-reducing bacteria are affected by sulfide stress, which might result in a change in their energy metabolism or induce ‘leakiness’ with regards to sulfur compounds with intermediate oxidation state. Sulfide toxicity also impacts organisms that can simultaneously perform methanogenesis and native sulfur reduction to sulfide. When grown with hydrogen and sulfur, methanogenesis by *Methanococcus thermolithotrophicus* ended before hydrogen sulfide production, resulting in cells lysis caused by sulfide toxicity (Stetter and Gaag, 1983).

Similar to sulfate-reducing bacteria, ANME that are involved in sulfate-driven AOM must be tolerant to sulfide. In some incubation experiments, AOM activity stopped at sulfide concentrations as low as 2.1 mM. Yet, this may have been caused by selection of weakly tolerant AOM cultures by sulfide removal during the culture enrichment (Meulepas et al., 2009). Other *in vitro* incubations operate at much higher sulfide concentrations, up to 14 mM (Nauhaus et al., 2005) and 15 mM (Wegener et al., 2016). Sulfide inhibition of AOM was observed for low sulfate concentrations (4 mM) and high sulfide concentrations (3–4 mM), but not with high sulfate (21 mM) and high sulfide (3–4 mM) concentrations, which implies that thermodynamics (energy yield) impact the sulfide tolerance of AOM (Timmers et al., 2015). Due to advection in methane seep environments, sulfate and methane availability is higher than in sulfate-methane transition zones in marine sediments that are governed by diffusion. Thus, AOM in seep environments is expected to show a higher sulfide tolerance, an interpretation that is supported by the observation of high sulfide concentrations of 10–15 mM in seep settings (Valentine, 2002; Joye et al., 2004) with extreme values of up to 40 mM (Vigneron et al., 2013, 2014). Considering that zero-valent sulfur has been observed in experiments with AOM (Milucka et al., 2012), the following questions arise: could ANME oxidize the toxic sulfide to native sulfur as a coping strategy, or alternatively, could this oxidation happen spontaneously once sulfide enters ANME cells, and what in either case would the electron acceptor be for such a reaction? A possibility is that sulfate-reducing bacteria, as in the case of methane oxidation by ANME (Scheller et al., 2016; Timmers et al., 2017), receive electrons from the methanotrophic archaea and sulfate acts as the electron acceptor. As a net reaction, this process corresponds to the comproportionation of sulfide and sulfate to form native sulfur, whereby the weakly exergonic AOM reaction may have to offset a slight potential energy loss by the comproportionation reaction under environmental conditions.

#### Salt Stress

In hypersaline solutions, cells must maintain the water activity of their cytoplasm higher than that of the surrounding brine to avoid loss of water (Csonka, 1989; Brown, 1990). Moreover, in order to generate cell turgor pressure, which is considered to be the driving force for cell extension, growth, and division, the cells need to maintain an intracellular osmotic pressure that is somewhat greater than that of the growth medium (Csonka, 1989; Brown, 1990; Oren, 1999; Welsh, 2000). Microbial strategies to cope with osmotic stress include maintenance of high intracellular salt concentrations and/or a range of low-molecular-weight organic solutes, including trehalose, glycine betaine, or glutamate, that are compatible with biological function of the cells (Brown, 1976; Oren, 1999; Welsh, 2000). Such organic compounds can either be taken up from the environment or synthesized by the organisms. Halophilic sulfate-reducing bacteria appear to have the ability to synthesize trehalose and to uptake glycine betaine, with the ability to accumulate glycine betaine from the environment being energetically more favorable than the synthesis of trehalose, which requires a significant investment of both energy and fixed carbon (Welsh et al., 1996). Bacterial sulfate reduction, including sulfate reduction coupled to AOM, is sustained at high salinities such as in deep hypersaline basins from the Mediterranean Sea (van der Wielen et al., 2005; van der Wielen and Heijs, 2007; Borin et al., 2009), Gulf of Mexico (Lloyd et al., 2006; Zhuang et al., 2016), Gulf of Cadiz (Maignien et al., 2013), in a hypersaline Dead Sea aquifer (Avrahamov et al., 2014), and the Great Salt Lake in Utah (Kjeldsen et al., 2007). A consequence of the energetically costly adaptation to osmotic stress is that with increasing salinity, metabolic reactions that yield little energy, such as the oxidation of acetate coupled to sulfate reduction, are no longer carried out (Oren, 1999). It can be speculated that in such cases, other organisms may use acetate. Candidates could be so far uncultivated bacterial phyla that have been widely detected in anaerobic environments (BD1-5, OP11, and OD1), who are likely to play important yet unrecognized roles in hydrogen production, sulfur cycling, and fermentation of refractory sedimentary carbon (Wrighton et al., 2012; Kantor et al., 2013). There is evidence that these organisms are present at hydrocarbon seeps and in deep-sea anoxic brine lakes (Pachiadaki et al., 2010; Antunes et al., 2011; Aoki et al., 2014). Acetoclastic methanogenesis is negatively impacted by high salinity (Waldron et al., 2007; Zhang et al., 2017). However, due to high salinity, glycine betaine and dimethylsulfoniopropionate (DMSP) and their degradation products trimethylamine and dimethyl sulfide (DMS) become available to microbes inhabiting hypersaline environments (King, 1984; Oren, 1990, 1999, 2008; Lai and Gunsalus, 1992; Welsh et al., 1996; Lai et al., 1999; Zhuang et al., 2011, 2016). Such methylated compounds serve as substrate for methanogenesis in hypersaline environments (Zhuang et al., 2016), which highlights the possibility that there is a link between the formation of zero-valent sulfur and carbon cycling via methanogenesis in hypersaline, sulfidic environments such as the Urania deep-sea basin.

### Feasibility of Native Sulfur Genesis in Absence of Classical Oxidants: Kinetic and Thermodynamic Considerations

It is critical to assess whether the proposed mechanisms for the genesis of native sulfur deposits from hydrocarbons and gypsum or anhydrite in the absence of classical oxidants is thermodynamically favorable. If this prerequisite is not a given for a considered mechanism, one has to invoke a coupling to another thermodynamically favorable process that would make the overall process feasible. Once native sulfur is formed and precipitated as a solid, the slow kinetics of sulfur dissolution may become a critical factor in its preservation. This could also be important when native sulfur is formed as an intermediate of a process as long as this intermediate is allowed to accumulate to a degree where it precipitates while the overall process remains thermodynamically feasible. The latter could be the case for organisms’ stress response, where survival becomes more important than energy gain maximization. Below, we show that there are numerous reactions that could yield native sulfur as a product. The proposed mechanisms do not represent individual pathways but overall net reactions, which may be carried out by individual or consortia of organisms. As such, the presented equations and thermodynamics only demonstrate the potential for the existence of a process, but do not give any indication about its actual presence.

#### Oxidation of Hydrocarbons Coupled to Sulfate Reduction to Native Sulfur

Sulfate reduction to native sulfur and concomitant conversion of gypsum into calcite as a bulk process is energetically favorable (Table 2). Examples calculated with glucose, acetate, and methane as organic substrates for environmental conditions demonstrate that genesis of native sulfur is (1) thermodynamically feasible, (2) with regards to energetic yield close, and in some cases superior to the genesis of sulfide, and (3) strongly pH-dependent, with low pH making native sulfur formation more attractive than sulfide generation (Table 2).

**Table 2:** Gibbs free energy yield for oxidation of hydrocarbons coupled to sulfate reduction.

Eq. #	Substrate	Product	Reaction consuming gypsum, yielding calcite	ΔG_0_′ kJ/mol substrate	ΔG_0_′ kJ/mol sulfur	ΔG_0_′ kJ/mol e^−^	Effect on pH −low/+high
Eq. T2.1	Glucose	Sulfide	C_6_H_12_O_6_ + 3CaSO_4_ ⋅ 2H_2_O →	−457.7	−152.6	−19.1	–
			3H_2_S + 3HCO_3_^−^ + 3CaCO_3_ + 3H^+^ + 6H_2_O				
Eq. T2.2		Native sulfur	C_6_H_12_O_6_ + 4CaSO_4_ ⋅ 2H_2_O →	−499.8	−125.0	−20.8	–
			4S^0^ + 2HCO_3_ + 4CaCO_3_ + 2H^+^ + 12H_2_O				
Eq. T2.3	Acetate	Sulfide	Na^+^ + CH_3_COO^−^ + CaSO_4_ ⋅ 2H_2_O →	−49.1	−49.1	−6.1	−
			Na^+^ + CaCO_3_ + HCO_3_^−^ + H_2_S + 2H_2_O				
Eq. T2.4		Native sulfur	3Na^+^ + 3CH_3_COO^−^ + 4CaSO_4_ ⋅ 2H_2_O + CO_2_ →	−61.5	−46.1	−7.7	−/+
			3Na^+^ + 4CaCO_3_ + 3HCO_3_^−^ + 4S^0^ + 11H_2_O				
Eq. T2.5	Methane	Sulfide	CH_4_ + CaSO_4_ ⋅ 2H_2_O →	−18.0	−18.0	−2.3	−
			H_2_S + CaCO_3_ + 3H_2_O				
Eq. T2.6		Native sulfur	3CH_4_ + 4CaSO_4_ ⋅ 2H_2_O + CO_2_ →	−30.5	−22.8	−2.9	+
			4S^0^ + 4CaCO_3_ + 14H_2_O				

#### Sulfur Comproportionation

Two reactions with inorganic compounds have been invoked for the genesis of native sulfur in epigenetic deposits. The first concept proposes a redox reaction that essentially does the opposite of a sulfur disproportionation reaction (Feely and Kulp, 1957) by combining a sulfur compound with a positive and a sulfur compound with a negative oxidation state to form zero-valent sulfur. This mechanism is referred to as synproportionation or comproportionation and can be described as the reaction between hydrogen sulfide and sulfuric acid:

(1) $$\begin{array}{l} 3{\text{H}}_{2}\text{S}+{\text{H}}_{2}{\text{SO}}_{4}\to 4{\text{S}}^{0}+4{\text{H}}_{2}\text{O}\\ \Delta {\text{G}}_{0}^{\prime }=-12.8\text{ kJ}/\mathrm{mol sulfur},\;-8.5\text{ kJ}/{\mathrm{mol e}}^{-} \end{array}$$

More relevant for an ENSD might be a formulation that uses gypsum rather than sulfuric acid:

(2) $$\begin{array}{l} 3{\text{H}}_{2}\text{S}+{\text{CaSO}}_{4}\cdot 2{\text{H}}_{2}\text{O}+{\text{CO}}_{2}\to 4{\text{S}}^{0}+{\text{CaCO}}_{3}+5{\text{H}}_{2}\text{O}\\ \Delta {\text{G}}_{0}^{\prime }=-9.3\text{ kJ}/\text{mol sulfur},\;-6.2\text{ kJ}/{\text{mol e}}^{-} \end{array}$$

This reaction is close to thermodynamic equilibrium and thus could proceed in either direction. So far, comproportionation has not been observed to be important at temperatures below 100°C but it may occur in association with thermochemical sulfate reduction (TSR) (Goldstein and Aizenshtat, 1994; Machel, 2001). Sulfide production by TSR is greatly accelerated in presence of native sulfur and sulfide (Goldstein and Aizenshtat, 1994; Machel, 2001), indicating that sulfur species with intermediate oxidation states play a critical role (Figure 3). Most large ENSDs display sulfur isotope signatures that indicate involvement of microbial sulfate reduction in the formation of sulfide or S^0^, which excludes comproportionation reactions coupled with TSR.

#### Coupling of Sulfide Oxidation to CO_2_ Reduction

A second proposed reaction is the oxidation of sulfide coupled to CO_2_ reduction by microorganisms, according to Parafiniuk et al. (1994):

(3) $$\begin{array}{l} 12{\text{H}}_{2}\text{S}+6{\text{CO}}_{2}\to 12{\text{S}}^{0}+{\text{C}}_{6}{\text{H}}_{12}{\text{O}}_{6}+6{\text{H}}_{2}\text{O}\\ \Delta {\text{G}}_{0}^{\prime }=+360.6\text{ kJ}/{\text{mol C}}_{6}{\text{H}}_{12}{\text{O}}_{6},+30.1\text{ kJ}/\text{mol sulfur},\\ \;+15.1\text{ kJ}/{\text{mol e}}^{-} \end{array}$$

This reaction is strongly endergonic, i.e., it requires energy, and is carried out by anoxygenic phototrophs that oxidize sulfide to native sulfur as a means to fix carbon (Brune, 1989; Friedrich et al., 2005). It is a process that could contribute to the genesis of syngenetic native sulfur deposits (for a review, see Ehrlich and Newman, 2009) if a yet unknown other energy source could substitute for light. It is important to notice that another reaction following the same pattern of coupling carbon dioxide reduction to sulfide oxidation is energetically more feasible in the absence of light; the conversion to methane:

(4) $$\begin{array}{l} 4{\text{H}}_{2}\text{S}+{\text{HCO}}_{3}{}^{-}+{\text{H}}^{+}\to {\text{CH}}_{4}+4{\text{S}}^{0}+3{\text{H}}_{2}\text{O},\\ \Delta {\text{G}}_{0}^{\prime }=-24.1\text{ kJ}/\text{mol methane},\;-6.0\text{ kJ}/\text{mol sulfur},\\ \;-3.0\text{ kJ}/{\text{mol e}}^{-} \end{array}$$

This reaction is strongly pH dependent, as well as dependent on the activities of the substrates and products of the reaction. To our knowledge, little research has been carried out in regard to the feasibility of this reaction. A coupling to the precipitation of carbonate minerals, which is promoted by the availability of calcium ions from the dissolution of calcium sulfate minerals, could provide a driving force for this process because the removal of carbonate ions is compensated by the genesis of CO_2_/carbonic acid:

(5) $$\begin{array}{l} {\text{Ca}}^{2+}+2{\text{HCO}}_{3}{}^{-}\to {\text{CaCO}}_{3}+{\text{CO}}_{2}+{\text{H}}_{2}\text{O},\\ \Delta {\text{G}}_{0}^{\prime }=-33.4\text{ kJ}/\text{mol calcite} \end{array}$$

This results in the overall reaction

(6) $$\begin{array}{l} {\text{Ca}}^{2+}+4{\text{H}}_{2}\text{S}+2{\text{HCO}}_{3}{}^{-}\to {\text{CaCO}}_{3}+{\text{CH}}_{4}+4{\text{S}}^{0}+3{\text{H}}_{2}\text{O},\\ \Delta {\text{G}}_{0}^{\prime }=-52.6\text{ kJ}/\text{mol calcite},\;-13.1\text{ kJ/mol sulfur},\\ \;-6.6{\text{ kJ/mol e}}^{-} \end{array}$$

This reaction could work in concert with sulfide production by methanotrophic sulfate reduction (AOM) coupled to the transformation of gypsum into carbonates (Eq. T2.5, Table 2). If the two reactions are coupled (yielding Eq. T2.6, Table 2), build-up of methane would not be observed because methane consumption coupled to sulfate reduction could out-pace methane production coupled to sulfide oxidation (Eq. 6), establishing a cryptic carbon cycle via methanogenesis.

#### Coupling of Sulfide Oxidation to Acetate Reduction

Acetate is a common product of incomplete sulfate reduction (Rabus et al., 2006) and methanogenic degradation of crude oil alkanes (Gray et al., 2010, 2011). It is also released during burial and moderate heating of sediments (up to 60°C; Wellsbury et al., 1997) and thermogenic cracking of kerogens (Lewan et al., 1979; Borgund and Barth, 1994). Indeed, ample presence of organic acids and their conjugated bases (i.e., anions) has been detected in deep basinal brines (Carothers and Kharaka, 1978; Surdam et al., 1984; Means and Hubbard, 1987; Giordano and Kharaka, 1994; Kharaka and Hanor, 2014). In oil field waters from Texas and California, organic acid anions contribute most of the total alkalinity at temperatures of 80–140°C; with acetate contributing more than 90% of the anions and reaching concentrations as high as 10 g/l (∼169 mM; Carothers and Kharaka, 1978; Surdam et al., 1984).

Acetate can be oxidized by dissimilatory sulfate reduction (e.g., Eqs. 2.3, 2.4, Table 2) or converted to methane by acetoclastic methanogenesis,

(7) $$\begin{array}{l} {\text{CH}}_{3}{\text{COOH}}^{-}+{\text{H}}_{2}\text{O}\to {\text{CH}}_{4}+{\text{HCO}}_{3}{}^{-},\\ \Delta {\text{G}}_{0}^{\prime }=-31.0\text{ kJ}/\text{mol acetate, methane},\;-15.5{\text{ kJ/mol e}}^{-}, \end{array}$$

or syntrophic methanogenesis, which couples syntrophic acetate oxidation

(8) $${\text{CH}}_{3}{\text{COOH}}^{-}+{\text{H}}^{+}+2{\text{H}}_{2}O\to 4{\text{H}}_{2}+2{\text{CO}}_{2},$$

to hydrogenotrophic methanogenesis:

(9) $$4{\text{H}}_{2}+{\text{CO}}_{2}\to {\text{CH}}_{4}+2{\text{H}}_{2}\text{O},$$

which yields the same net reaction as acetoclastic methanogenesis (Eq. 7). At high carbon dioxide concentrations in oil reservoirs, acetoclastic methanogenesis appears to dominate over syntrophic methanogenesis (Mayumi et al., 2013). Syntrophic methanogenesis appears to be the dominant acetate degradation pathway once sulfate is depleted beneath the seabed (Beulig et al., 2018a), whereas it has also been shown that acetoclastic methanogenesis can take place in the presence of sulfate (Ozuolmez et al., 2015; Lv et al., 2015, 2016). The low energy yields for acetate oxidation by dissimilatory sulfate reduction (Eq. 2.3, 2.4, Table 2) and methanogenesis (Eq. 7) are likely the reasons why these reactions become inhibited under salt stress (Oren, 1999; Waldron et al., 2007; Zhang et al., 2017). Interestingly, the reaction for methanogenesis (Eq. 7) can be combined with carbon dioxide reduction to methane coupled to the oxidation of sulfide to native sulfur (Eq. 4), yielding exergonic reactions for native sulfur generation combined with methanogenesis with acetate as reactant:

(10) $$\begin{array}{l} {\text{CH}}_{{}_{3}}{\text{COOH}}^{-}+{\text{H}}^{+}+4{\text{H}}_{2}\text{S}\to 2{\text{CH}}_{4}+4{\text{S}}^{0}+2{\text{H}}_{2}\text{O},\\ \Delta {\text{G}}_{0}^{\prime }=-55.1\text{ kJ}/\text{mol acetate},\;-27.6\text{ kJ}/\text{mol methane},\\ \;-13.8\text{ kJ}/\text{mol sulfur},\;-6.9\text{ kJ}/{\text{mol e}}^{-} \end{array}$$

This process is energetically attractive, which could render it more feasible under saline conditions. Also, it effectively copes with sulfide stress because it removes four moles of sulfide per mole acetate and simultaneously drives the pH to more basic conditions, shifting sulfide speciation from H_2_S to the bisulfide ion, which does not diffuse through cell membranes.

#### Coupling of Sulfide Oxidation to Native Sulfur to Methanogenesis

Based on the observation that hypersaline, sulfidic environments such as the Urania deep-sea basin may be a location where sulfur with intermediate oxidation state is produced, it is interesting to explore the thermodynamics of such coupled sulfur-carbon cycling. Methylated substrates such as methanol, which is derived from lignin and pectin degradation (Donnelly and Dagley, 1980; Schink and Zeikus, 1980), trimethylamine, and DMS serve as substrate for methanogenesis in hypersaline environments (Zhuang et al., 2016):

Methanol-based methanogenesis:

(11) $$\begin{array}{l} 4{\text{CH}}_{{}_{3}}\text{OH}\to 3{\text{CH}}_{4}+{\text{HCO}}_{3}{}^{-}+{\text{H}}^{+}+{\text{H}}_{2}\text{O},\\ \Delta {\text{G}}_{0}^{\prime }=-78.6\text{ kJ}/\text{mol methanol},\;-104.8\text{ kJ}/\text{mol methane},\\ \;-52.4\text{ kJ}/{\text{mol e}}^{-} \end{array}$$

Dimethyl sulfide-based methanogenesis:

(12) $$\begin{array}{l} 2{\left({\text{CH}}_{{}_{3}}\right)}_{2}\text{S}+3{\text{H}}_{2}\text{O}\to 3{\text{CH}}_{4}+{\text{HCO}}_{3}{}^{-}+2{\text{H}}_{2}\text{S}+{\text{H}}^{+},\\ \Delta {\text{G}}_{0}^{\prime }=+3.9\text{ kJ}/\text{mol DMS},\;+2.6\text{ kJ}/\text{mol methane},\\ \;+1.3\text{ kJ}/{\text{mol e}}^{-} \end{array}$$

Trimethylamine-based methanogenesis

(13) $$\begin{array}{l} 4{\left({\text{CH}}_{{}_{3}}\right)}_{3}{\text{NH}}^{+}+9{\text{H}}_{2}O\to 9{\text{CH}}_{4}+3{\text{HCO}}_{3}{}^{-}+4{\text{NH}}_{4}{}^{+}+3{\text{H}}^{+},\\ \Delta {\text{G}}_{0}^{\prime }=-105.9\text{ kJ}/\text{mol }{\left({\text{CH}}_{{}_{3}}\right)}_{3}{\text{NH}}^{+},\;-47.1\text{ kJ}/\text{mol methane},\\ \;-23.5\text{ kJ}/{\text{mol e}}^{-} \end{array}$$

These reactions can be combined with the process of carbon dioxide reduction to methane coupled to the oxidation of sulfide to native sulfur (Eq. 4), yielding exergonic reactions for native sulfur generation combined with methanogenesis.

Methanol-based methanogenesis:

(14) $$\begin{array}{l} {\text{H}}_{2}\text{S}+{\text{CH}}_{3}\text{OH}\to {\text{CH}}_{4}+{\text{S}}^{0}+{\text{H}}_{2}\text{O},\\ \Delta {\text{G}}_{0}^{\prime }=-84.7\text{ kJ}/\text{mol methanol, methane, sulfur,}\\ \;-42.3\text{ kJ}/{\text{mol e}}^{-} \end{array}$$

Dimethyl sulfide-based methanogenesis:

(15) $$\begin{array}{l} 2{\text{H}}_{2}\text{S}+2{\left({\text{CH}}_{3}\right)}_{2}\text{S}\to 4{\text{CH}}_{4}+4{\text{S}}^{0},\\ \Delta {\text{G}}_{0}^{\prime }=-139.1\text{ kJ}/\text{mol DMS, }-69.6\text{ kJ}/\text{mol methane, sulfur,}\\ \;-34.8\text{ kJ}/{\text{mol e}}^{-} \end{array}$$

Trimethylamine-based methanogenesis

(16) $$\begin{array}{l} 3{\text{H}}_{2}\text{S}+{\left({\text{CH}}_{3}\right)}_{3}NH^{+}\to 3{\text{CH}}_{4}+4{\text{S}}^{0}+NH_{4}{}^{+},\\ \Delta {\text{G}}_{0}^{\prime }=-124.0\text{ kJ}/\text{mol }{\left({\text{CH}}_{3}\right)}_{3}NH^{+},\;-41.3\text{ kJ}/\text{mol methane},\\ \;-31.0\text{ kJ}/\text{mol sulfur},\;-20.7\text{ kJ}/{\text{mol e}}^{-} \end{array}$$

This demonstrates that under sulfidic conditions in a hypersaline environment, such reactions would be favorable, which would be compatible with the observations from the Urania deep basin, where methanogenesis greatly exceeds sulfate reduction, and sulfur cycling appears to utilize sulfur compounds with intermediate oxidation states (Borin et al., 2009).

#### Coupling of Sulfate Reduction With Ammonium Oxidation and Sulfide Oxidation With N_2_ Reduction

Another possibility of native sulfur generation has been proposed based on observations from wastewater treatment. Sulfur and nitrogen mass balance considerations indicate that during the removal of total Kjeldahl nitrogen (TKN), sulfate reduction to native sulfur via coupling to anaerobic ammonium oxidation yields dinitrogen gas (N_2_), a reaction that is exergonic at standard conditions (Fdz-Polanco et al., 2001).

(17) $$\begin{array}{l} 2{\text{NH}}_{4}{}^{+}+{\text{SO}}_{4}{}^{2-}\to {\text{N}}_{2}+{\text{S}}^{0}+4H_{2}O,\\ \Delta {\text{G}}_{0}^{\prime }=-22.7\text{ kJ}/\text{mol ammonium},\;-45.5\text{ kJ}/\text{mol sulfur},\\ \;-7.6\text{ kJ}/{\text{mol e}}^{-} \end{array}$$

At standard conditions at pH 7, the reaction to native sulfur is energetically equivalent to the production of sulfide and N_2_ from sulfate and ammonium, which becomes favorable at high pH (Schrum et al., 2009).

(18) $$\begin{array}{l} 8{\text{NH}}_{4}{}^{+}+3{\text{SO}}_{4}{}^{2-}\to 4{\text{N}}_{2}+3{\text{H}}_{2}S+12H_{2}O+5H^{+},\\ \Delta {\text{G}}_{0}^{\prime }=-17.6\text{ kJ}/\text{mol ammonium},\;-46.9\text{ kJ}/\text{mol sulfur},\\ \;-5.9\text{ kJ}/{\text{mol e}}^{-} \end{array}$$

To further explore coupled nitrogen-sulfur cycling, and in analogy to the coupling of sulfide oxidation with reduction of carbon dioxide to produce native sulfur and methane (Eq. 4), one can consider a coupling of sulfide oxidation to native sulfur with the reduction of dinitrogen gas (N_2_) to ammonium.

(19) $$\begin{array}{l} 3{\text{H}}_{2}\text{S}+{\text{N}}_{2}+2{\text{H}}_{2}\text{O}\to 2{\text{NH}}_{4}{}^{+}+3{\text{S}}^{0}+2{\text{OH}}^{-},\\ \Delta {\text{G}}_{0}^{\prime }=+4.7\text{ kJ}/\text{mol ammonium},\;+2.3\text{ kJ}/\text{mol sulfur},\\ \;+1.6\text{ kJ}/{\text{mol e}}^{-} \end{array}$$

The conversion of sulfide and N_2_ into ammonium and native sulfur is endergonic at standard conditions at neutral pH. For the genesis of native sulfur via N_2_ reduction to ammonium, a low pH is favorable. Addition of the two equations (Eq. 18 and 19) yields the equation for the genesis of native sulfur from ammonium and sulfate (Eqs. 17). Crude oil contains nitrogen as a component of organic molecules (e.g., Qian et al., 2001; Shi et al., 2010; Prado et al., 2017), and ammonia is present in fluids in sedimentary basins (Kharaka and Hanor, 2014). There are processes that release and sequester nitrogenous compounds from and into organic compounds as well as minerals (Lindgreen, 1994; Schimmelmann and Lis, 2010). Thus, from a quantitative perspective, it is not certain if coupled nitrogen-sulfur cycling could be responsible for the formation of ENSDs. Aside from this caveat, it is noteworthy that the reactions involving the genesis or consumption of ammonium (a weak acid) can strongly impact the pH (Eqs. 17–19). This characteristic could serve as a means to maintain the pH of the environment in a favorable range. In a sulfide-stressed setting, where the sulfide speciation is critical because neutrally charged H_2_S easily diffuses through cell membranes (Barton et al., 2014), genesis of native sulfur and shifting the pH to higher values (Eq. 19, and to a lesser degree Eq. 17) could be an effective strategy to cope with sulfide stress.

#### Thermodynamic Feasibility – A Summary

Genesis of native sulfur is thermodynamically feasible when linked to hydrocarbon (including methane) oxidation, sulfur comproportionation, methanogenesis, and coupling to nitrogen cycling. Considering the generally low energy yields, it is evident that *in situ* conditions, particularly the actual activities and fugacities of the compounds involved in the reactions are decisive if individual reactions are favorable. Nevertheless, from the wide variety of potential processes, it follows that there is no thermodynamic argument that would exclude genesis of native sulfur in an anoxic setting as a viable option.

## Hypothesis

We put forward the concept that the genesis of ENSDs takes place in saline to hypersaline, highly sulfidic environments that are devoid of external oxidants such as O_2_, nitrate, metal oxides or light. Salinity and sulfide stress result in a setting in which genesis of native sulfur is caused by one or more of the four processes:

(1) Bacterial sulfate reducers release sulfur compounds with intermediate oxidation state, which are then further metabolized to zero-valent sulfur,
(2) Bacterial sulfate reduction ceases to metabolize acetate, which enables yet uncultivated microorganisms to establish a sulfur cycle that produces zero-valent sulfur,
(3) Methanogens – potentially in syntrophic partnership with other organisms – couple methylotrophic methane production or carbon dioxide and acetate reduction to the oxidation of sulfide to zero-valent sulfur,
(4) ANME engage in the oxidation of sulfide to zero-valent sulfur, potentially by transferring electrons (Scheller et al., 2016; Skennerton et al., 2017) to their sulfate-reducing partners.

In these scenarios, native sulfur is preserved because the high levels of sulfide inhibit further disproportionation of zero-valent sulfur, leading to the accumulation of dissolved zero-valent sulfur and polysulfides and the subsequent precipitation of native sulfur (Figure 3). Formation of native sulfur could be further modulated by coupling of sulfur transformations to nitrogen cycling as a means to cope with sulfide stress.

There are two major reasons why such processes may have eluded detection so far:

(1) Their detection in the environment is hampered by poor accessibility of sites where they may occur, and due to the challenge that any *in situ* analysis is invasive and may introduce O_2_, thereby creating potential artifacts.
(2) Their detection in the laboratory is hampered by experimental challenges, including O_2_-contamination at all times, working with high pressures (e.g., methane, CO_2_) and high concentrations of sulfide and/or salt over long durations. The latter may further slow down a process with a notoriously low energy yield due to salt and sulfide stress.

## Conclusion and Outlook

Several lines of evidence indicate that the genesis of native sulfur in ENSDs has occurred in absence of O_2_. This does not preclude the genesis of native sulfur by oxidation with O_2_, as there are examples where that option appears realistic, such as in intermittent oxidation of sulfide (Jassim et al., 1999) or the oxidation of sulfide in a soil-influenced environment (Peckmann et al., 1999). In analogy to the genesis of caves (Kirkland, 2014), large cavities in carbonate caprocks from the United States Gulf Coast (Fenneman, 1906; Barton and Paxson, 1925; Taylor, 1938), could be due to leaching by sulfuric acid formed by the oxidation of native sulfur with O_2_. Acid generation could even cause spontaneous and rapid native sulfur precipitation from polysulfides. While acknowledging that such scenarios exist, our hypothesis removes the dogma that O_2_ must be available for the genesis of large amounts of native sulfur. We show that formation of native sulfur in strictly anoxic environments is not only thermodynamically feasible, but also that highly saline and sulfidic conditions are conducive for a suite of microbial processes to occur that could yield zero-valent sulfur as product (Figure 3). The hypothesized microbial pathways are compatible with so far puzzling observations, like the persistent presence of sulfur disproportionating organisms in AOM cultures and in the environment (Wegener et al., 2016), the ubiquity of the *Deltaproteobacteria* despite low sulfate reduction rates in hypersaline waters (Borin et al., 2009), and the apparent presence of oxidative (cryptic) sulfur cycling near sulfate-methane transition zones (Holmkvist et al., 2011; Brunner et al., 2016). Moreover, they could serve as a model for the genesis of (1) native sulfur nodules that are infrequently found in drill cores in sediments with little iron content, which allows for high sulfide concentrations, such as sediments from the Bahamas (Bahamas Transect ODP Leg 166, Site 1005, Hole C, Core 033; Shipboard Scientific Party, 1997) and the Great Australian Bight (Leg 182, Site 1129C, Core 20H, Section 4; Feary et al., 2000), which is a carbonate sequence in which sulfide and methane are co-generated (Mitterer et al., 2001), or Lake Petén Itzá (Core 6A-4H-2; Hodell et al., 2006); and (2) native sulfur associated with hydrocarbon seep environments (Lin et al., 2018).

Examples for which there are indications for the genesis of native sulfur in the absence of a classical oxidant include sulfur transformations by AOM and sulfur-carbon cycling in the highly sulfidic, hypersaline Urania deep-sea basin. Moreover, our thermodynamic calculations imply that genesis of native sulfur may be coupled to methanogenic pathways that couple oxidation of sulfide to the reduction of carbon dioxide or acetate (Eqs. 4, 10). From this follows that methane cycling may play a critical role in the genesis of ENSDs. However, this does not imply that the here proposed mechanisms are only applicable to systems that are fueled by methane (natural gas), but not to native sulfur deposits that are fueled by crude oil. Methanogenesis is a key process in the formation of heavy oil and can take place in the presence of sulfate (for a review, see Gray et al., 2010). However, in a sulfate-rich environment, it is likely that the produced methane is immediately consumed by AOM coupled to sulfate reduction. Thus, genesis of ENSDs in an oil-dominated system can take place, even if the process is tied to methane transformations that remain hidden. Such cryptic carbon cycling via methane has been identified at the sulfate-methane transition in marine sediments (Beulig et al., 2018b), notably the very same environment for which cryptic sulfur cycling has been inferred (Holmkvist et al., 2011; Brunner et al., 2016). If the genesis of native sulfur and methane is coupled (Eqs. 4, 10), the coincidence of cryptic carbon and sulfur cycles at the sulfate-methane transition would be a logical consequence: disproportionation of native sulfur to sulfide and sulfate provides the oxidant for subsequent methane oxidation by AOM. In ENSDs, sulfur disproportionation would not take place because the reaction is unfavorable at high sulfide concentrations, allowing for the accumulation of native sulfur.

Multiple avenues of research can be taken to test our hypothesis. Long-term anaerobic incubations of mixed cultures obtained from sediments from seeps and hypersaline lakes may yield native sulfur – or should accumulate sulfide to a level where metabolic activity ceases. Amendments of such incubations with methylated substrates or acetate could be used to test if formation of native sulfur can be accelerated, a step that might be critical because metabolic rates are expected to be slow due to low energy yields of the involved reactions. The recent findings that dissimilatory sulfur cycling can be carried out by a much larger diversity of microbial groups than previously thought (Rückert, 2016; Anantharaman et al., 2018), including rice paddy *Nitrospirae* (Zecchin et al., 2018), also enhance the potential to find a dissimilatory sulfate reduction pathway that may yield a sulfur compound with intermediate oxidation state instead of sulfide. The fact that the group of peatland *Acidobacteria*, which only possess the genomic toolset to convert sulfite into sulfide and encodes enzymes that liberate sulfite from organosulfonates (Hausmann et al., 2018), also points to the possibility that alternative sulfate reduction pathways may exist. Such pathways could resemble assimilatory sulfate reduction, which is employed by a much wider group of microorganisms than dissimilatory sulfate reduction (Peck, 1961). *Acidobacteria* that have the ability to perform oxidative and reductive sulfur cycling (Hausmann et al., 2018) might be a particularly interesting target in sulfur cycling in ENSDs. They are adapted to acidic conditions, which can exist in fluids in sedimentary basins (Kharaka and Hanor, 2014) and have been found in soils and aquifers contaminated with hydrocarbons (Coates et al., 1999; Salam et al., 2017), which indicates that they can tolerate exposure to oil. Their versatility, and particularly ability to thrive at low abundance of nutrients while showing high tolerance to toxic compounds, may give *Acidobacteria* competitive advantages (Kielak et al., 2016) in settings that are nutrient-poor (evaporite rocks), rich in hydrocarbons, and have elevated concentrations of toxic compounds. For two reasons, *Acidobacteria* may represent ideal candidates to challenge our hypothesis that native sulfur genesis takes place in the absence of an external oxidant. (1) Many *Acidobacteria* are aerobes or facultative anaerobes (Kielak et al., 2016; Hausmann et al., 2018), which might enable genesis of native sulfur in presence of O_2_. (2) Their adaptation to acidic conditions could be advantageous in native sulfur formation that is driven by dynamic changes in hydrocarbon and O_2_ supply, where the pH might fluctuate between acidic conditions due to supply with acidic deep fluids, which can exist in sedimentary basins (Kharaka and Hanor, 2014), and neutral to basic conditions during which carbonates precipitate.

A suite of geological and geochemical analyses could be employed to explore our hypothesis that native sulfur genesis takes place in the absence of an external oxidant. Although morphological evidence for their presence is difficult to find in rocks, biofilms have been involved in seep carbonate formation (Hagemann et al., 2013; Zwicker et al., 2018), implying that they might have been overlooked in authigenic carbonates associated with ENSDs. Searching for fabrics that may represent former biofilms and analyzing them for trace metals and biomarkers could reveal if there were steep biogeochemical gradients during carbonate formation, which may be indicative for the presence of electron acceptors and sulfide in close spatial or temporal proximity. Biomarkers of sulfate-reducing bacteria and methanotrophic archaea involved in anaerobic oxidation of methane (Peckmann and Thiel, 2004) could provide insight if cryptic carbon cycling includes not only methane consumption but also methanogenesis with both processes being potentially involved in the formation of ENSDs fueled by crude oil. To test if native sulfur was formed by comproportionation under high temperatures, carbonates from native sulfur deposits can be analyzed for their clumped carbon-oxygen isotope composition to pin down the temperature of carbonate formation or diagenesis (e.g., Ghosh et al., 2006; Kele et al., 2015; Millán et al., 2016), a measurement that can be combined with the extraction of tourmaline formed in the early diagenesis of evaporites, which is a recorder of their thermal history (Henry et al., 1999; Henry and Dutrow, 2012). Finally, high-resolution sulfur isotope analysis with multicollector-ICPMS (e.g., Craddock et al., 2008; Paris et al., 2013; Present et al., 2015) or SIMS (e.g., Richardson et al., 2016; Gomes et al., 2018) techniques of CAS and other sulfur phases will reveal if the sulfur isotope systematics follow steady-state or Rayleigh fractionation patterns. With these approaches, we now have the tools to solve a conundrum that has puzzled scientists for over 100 years.

## Author Contributions

AL and BB designed the study. AL, BB, and SB analyzed the samples. All authors contributed to the interpretation of data and the writing of the manuscript.

## Conflict of Interest Statement

The authors declare that the research was conducted in the absence of any commercial or financial relationships that could be construed as a potential conflict of interest.
